# Transcription factors HB21/40/53 trigger inflorescence arrest through abscisic acid accumulation at the end of flowering

**DOI:** 10.1093/plphys/kiae234

**Published:** 2024-04-26

**Authors:** Verónica Sánchez-Gerschon, Irene Martínez-Fernández, María R González-Bermúdez, Sergio de la Hoz-Rodríguez, Florenci V González, Jorge Lozano-Juste, Cristina Ferrándiz, Vicente Balanzà

**Affiliations:** Instituto de Biología Molecular y Celular de Plantas, Consejo Superior de Investigaciones Científicas-Universitat Politècnica de Valencia, 46022 Valencia, Spain; Instituto de Biología Molecular y Celular de Plantas, Consejo Superior de Investigaciones Científicas-Universitat Politècnica de Valencia, 46022 Valencia, Spain; Instituto de Biología Molecular y Celular de Plantas, Consejo Superior de Investigaciones Científicas-Universitat Politècnica de Valencia, 46022 Valencia, Spain; Departament de química inorgànica i orgànica, Universitat Jaume I, 12071 Castelló, Spain; Departament de química inorgànica i orgànica, Universitat Jaume I, 12071 Castelló, Spain; Instituto de Biología Molecular y Celular de Plantas, Consejo Superior de Investigaciones Científicas-Universitat Politècnica de Valencia, 46022 Valencia, Spain; Instituto de Biología Molecular y Celular de Plantas, Consejo Superior de Investigaciones Científicas-Universitat Politècnica de Valencia, 46022 Valencia, Spain; Instituto de Biología Molecular y Celular de Plantas, Consejo Superior de Investigaciones Científicas-Universitat Politècnica de Valencia, 46022 Valencia, Spain

## Abstract

Flowers, and hence, fruits and seeds, are produced by the activity of the inflorescence meristem after the floral transition. In plants with indeterminate inflorescences, the final number of flowers produced by the inflorescence meristem is determined by the length of the flowering period, which ends with inflorescence arrest. Inflorescence arrest depends on many different factors, such as the presence of seeds, the influence of the environment, or endogenous factors such as phytohormone levels and age, which modulate inflorescence meristem activity. The FRUITFULL-APETALA2 (FUL-AP2) pathway plays a major role in regulating the end of flowering, likely integrating both endogenous cues and those related to seed formation. Among AP2 targets, *HOMEOBOX PROTEIN21* (*HB21*) has been identified as a putative mediator of AP2 function in the control of inflorescence arrest. HB21 is a homeodomain leucine zipper transcription factor involved in establishing axillary bud dormancy. Here, we characterized the role of HB21 in the control of the inflorescence arrest at the end of flowering in Arabidopsis (*Arabidopsis thaliana*). *HB21*, together with *HB40* and *HB53*, are upregulated in the inflorescence apex at the end of flowering, promoting floral bud arrest. We also show that abscisic acid (ABA) accumulation occurs in the inflorescence apex in an *HB*-dependent manner. Our work suggests a physiological role of ABA in floral bud arrest at the end of flowering, pointing to ABA as a regulator of inflorescence arrest downstream of the *HB21/40/53* genes.

## Teaser

Three homeodomain leucine zipper transcription factors trigger abscisic acid accumulation in the inflorescence apex at the end of the reproductive phase, arresting unpollinated floral bud development.

## Introduction

The flowering period is the time during which a plant produces flowers. It initiates with the floral transition, where the shoot apical meristems (SAMs) acquire inflorescence identity, in a highly regulated developmental process that integrates multiple signals, both endogenous and exogenous (Andrés and Coupland 2012; Blümel et al. 2015; Kinoshita and Richter 2020; Freytes et al. 2021). The inflorescence meristem, then, actively generates flowers for a period of time, until its arrest and the concomitant cessation of flower opening. Despite its ecological and agronomical interest, the end of flowering is still a largely uncharacterized process, even though our knowledge on the topic is increasing rapidly in the last years. These recent studies suggest that the end of flowering caused by inflorescence arrest, similarly to the floral transition, is a complex developmental process (González-Suárez et al. 2020; Wang et al. 2022; Balanzà et al. 2023).

The inflorescence behavior at the end of the flowering phase can be divided into 2 different components: the proliferative arrest of the inflorescence meristem, which becomes inactive and stops producing new flower primordia, and the developmental block of the unpollinated floral buds already produced around the moment of meristem arrest, also known as floral arrest (Walker et al. 2023). In Arabidopsis (*Arabidopsis thaliana*), these combined events produce the typical morphology associated with the end of the flowering phase, where a defined cluster of unopened floral buds remains at the apex of the arrested inflorescence. At the genetic level, the proliferative arrest of the inflorescence meristem is controlled by the FRUITFULL-APETALA2 (FUL-AP2) pathway that has been proposed to integrate age-dependent and other endogenous signals (Balanzà et al. 2018). The AP2 transcription factor promotes meristem activity maintaining the expression of *WUSCHEL* (*WUS*), a stem cell identity gene (Laux et al. 1996; Mayer et al. 1998; Wurschum et al. 2006; Zhao et al. 2007). The MADS-box transcription factor FUL promotes the end of flowering, in part, by the direct repression of the *AP2* gene and other members of the *AP2* clade (Balanzà et al. 2018), which are also negatively regulated by miR172. Thus, while *ap2* mutant combinations with other mutants of the *AP2* family show a shorter flowering period, the *AP2* alleles resistant to miR172 cause a delayed meristem arrest, and *ful* mutants do not cease meristem activity and are able to produce flowers until the death of the plant (Balanzà et al. 2018; Merelo et al. 2022). Cytokinins (CKs) have been related to the control of inflorescence meristem activity. It has been shown that before meristem arrest at the end of flowering, the CK responses decrease in the inflorescence meristem, being completely blocked at the moment of arrest (Merelo et al. 2022; Walker et al. 2023). The decrease in CK response is associated with a decrease in cell division rate and with the decline in the expression of *WUS*, also absent at the moment of meristem arrest (Merelo et al. 2022). Interestingly, it has been shown that AP2 represses several negative regulators of CK signaling, thus promoting CK responses in the meristem (Martínez-Fernández et al. 2020). Additionally, environmental factors as temperature or light quality have been also proposed as modulators of this developmental process (Martínez-Fernández et al. 2020; González-Suárez et al. 2023). Together with this genetic, hormonal, and environmental control, a major factor controlling the end of flowering is seed production, which acts as a strong inflorescence arrest promoter. In sterile mutants, or in plants where fruit and seed production is prevented, the inflorescence meristem remains active for longer, ending the flowering period with the differentiation of the SAM into a terminal floral structure (Hensel et al. 1994; Balanzà et al. 2019).

The factors described so far have been mainly related with the regulation of the activity of the inflorescence meristem arrest at the end of the flowering period. However, how the floral arrest is established is poorly understood. It was described that auxin export from developing fruits could trigger inflorescence arrest by mechanisms that were still unclear (Ware et al. 2020; Goetz et al. 2021). A recent work restricts this role of auxin to the floral bud arrest observed at the end of flowering rather than to the control of the cessation of inflorescence meristem activity (Walker et al. 2023). CK also seems to play a role in the floral arrest, as mutants with increased CK sensitivity show reduced floral bud clusters compared to control plants (Walker et al. 2023).

The inflorescence meristem arrest at the end of the flowering phase has been interpreted as a dormancy state. Transcriptomic profiles of arrested meristems at the end of flowering show a high degree of similarity with those of dormant meristems, presenting low mitotic activity and the activation of responses related to stress and growth inhibitory hormones such as the abscisic acid (ABA; Wuest et al. 2016). In agreement with this, meristem arrest at the end of flowering can be reverted by seed/fruit removal, *AP2* induction, or CK treatments (Hensel et al. 1994; Balanzà et al. 2018; Merelo et al. 2022).

Axillary bud dormancy in Arabidopsis is controlled by the TCP transcription factor *BRANCHED1* (*BRC1*), which promotes cell growth arrest, preventing the activation of the axillary meristems (Aguilar-Martínez et al. 2007). ABA signaling is one of the growth arrest responses controlled by BRC1 through the activation of 3 related homeodomain leucine zipper (HD-ZIP) transcription factors: *HOMEOBOX PROTEIN21* (*HB21*), *HB53*, and *HB40*. These 3 factors upregulate *9-CIS-EPOXICAROTENOID DIOXIGENASE 3* (*NCED3*), a key gene in the ABA biosynthesis pathway (Iuchi et al. 2001; Tan et al. 2003), thus triggering ABA accumulation (González-Grandío et al. 2017). While the role of ABA in axillary bud dormancy is well established (Yao and Finlayson 2015; González-Grandío et al. 2017; van Es et al. 2024), its possible role in the regulation of inflorescence arrest at the end of flowering is essentially unknown. Recently, we have identified several genes involved in the control of axillary bud dormancy that are also downstream the FUL-AP2 pathway that controls inflorescence proliferative arrest. AP2 is a direct repressor of *HB21*, and when *AP2* is induced in active inflorescence apexes, the levels of *HB21*, *HB53*, and, in a lesser extent, *HB40* are reduced, together with the ABA responses associated to the end of flowering and meristem arrest (Yant et al. 2010; Martínez-Fernández et al. 2020).

In this work, we have characterized the expression of *HB21* during inflorescence development, as well as the role of *HB21*, *HB53*, and *HB40* at the end of flowering. We show that the *HB21*, *HB53*, and *HB40* genes act redundantly to promote the floral arrest associated to the end of flowering. Transcriptomic analyses also indicate that the induction of *HB21* in young apexes promotes similar responses to the observed in arrested inflorescences or dormant axillary buds, mainly through the regulation of ABA accumulation and responses. Finally, our work indicates that ABA is a key regulator of the floral arrest at the end of flowering, acting downstream of *HB21/40/53* genes and incorporating ABA as a player in the control of inflorescence arrest.

## Results

### HB21 accumulates in the inflorescence apex close to the end of flowering

The role of *HB21*, together with *HB40* and *HB53*, in the maintenance of axillary bud dormancy in Arabidopsis has been previously described (González-Grandío et al. 2017). Interestingly, it has also been suggested that *HB21* could be involved in the control of the end of flowering mediating the inflorescence meristem arrest, which has been proposed to be a type of meristem dormancy (Wuest et al. 2016; Martínez-Fernández et al. 2020). However, the relevance of *HB21* during this developmental process is still unclear.

**Figure 1.**
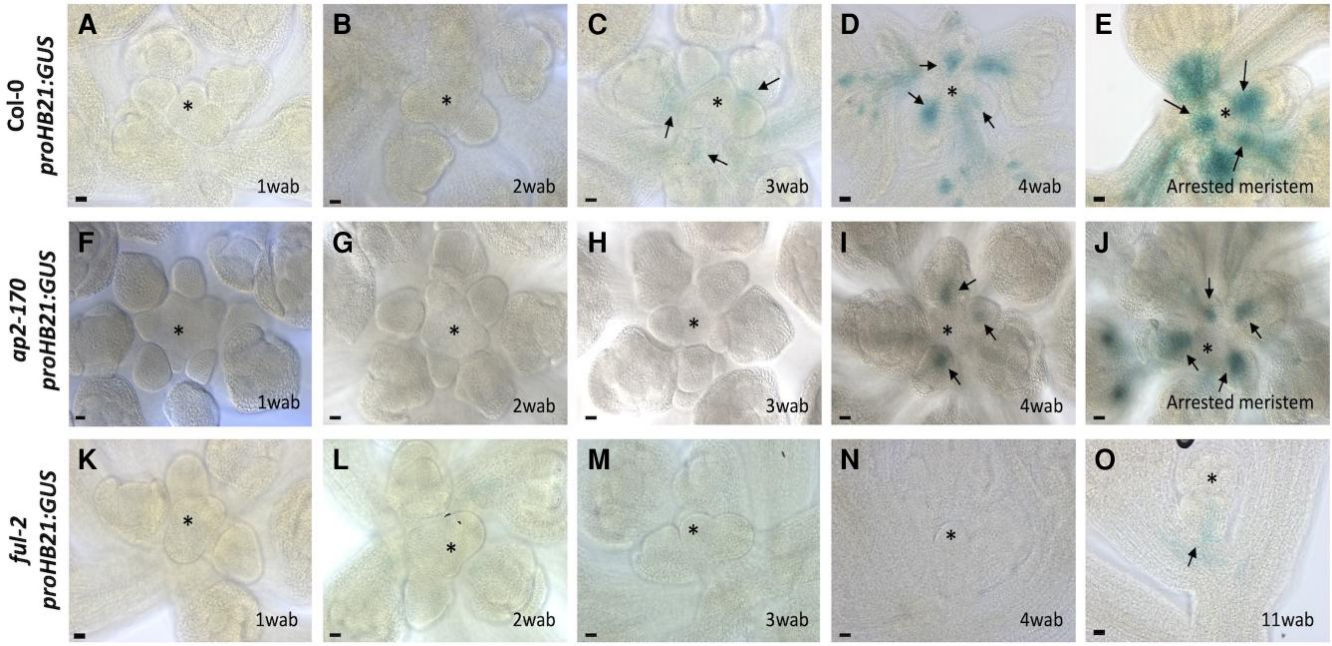
HB21 expression pattern accumulates close to the end of flowering. Histochemical detection of GUS activity driven by the *HB21* promoter in Arabidopsis inflorescence apex. **A to E)** Expression of pattern *proHB21:GUS* in WT background 1 wab **A)**, 2 wab **B)**, 3 wab, at the onset of the low proliferative phase, when weak activity of the reporter is detected **C)**, 4 wab, when upregulation of the reporter is clear **D)**, and in arrested meristems, where the HB21 expression reaches a maximum **E)**. **F to J)** Expression pattern of *proHB21:GUS* in the *ap2-170* background 1 wab **F)**, 2 wab **G)**, 3 wab **H)**, 4 wab, when the *HB21* expression begins **I)**, and in arrested meristems **J)**. **K to O)** Expression pattern of *proHB21:GUS* in *ful-2* background 1 wab **K)**, 2 wab **L)**, 3 wab **M)**, 4 wab **N)**, and at 11 wab, where the plant is entering senescence, a slight *HB21* expression can be detected **O)**. Arrowheads point to the floral buds. Asterisk marks the SAM. Bars represent 20 *µ*m.

To understand the role of *HB21* during inflorescence arrest, we decided to study its expression pattern in the inflorescence apex during the entire flowering period using a *proHB21:GUS* reporter line (González-Grandío et al. 2017). The GUS signal was absent in the inflorescence apex 1 wk after bolting (wab; Fig. 1A), as well as at 2 wab (Fig. 1B). At 3 wab, when the proliferative capacity of inflorescence meristem declines (Merelo et al. 2022), the GUS signal started to be detected in the base of the floral buds at the inflorescence apex (Fig. 1C), being evident at 4 wab, when the meristem is arrested (Fig. 1, D and E). Interestingly, the GUS signal was restricted to floral buds and never detected in the SAM itself (Fig. 1, A to E).

To determine that the expression detected in the apex was related to the developmental arrest of the SAM and not a mere temporal correlation with inflorescence age, we decided to introduce the *proHB21:GUS* reporter in the *ap2-170* and *ful-2* mutant backgrounds, in which the activity of the inflorescence SAM is extended. *ap2-170* is a miR172-resistant allele that presents a delayed meristem arrest caused by enhanced AP2 accumulation (Balanzà et al. 2018). It has been shown that AP2 is a direct negative regulator of *HB21* (Yant et al. 2010; Martínez-Fernández et al. 2020). The *proHB21:GUS* reporter in the *ap2-170* mutant was not detected during the first 3 wab (Fig. 1, F to H). The GUS signal appeared at low levels at 4 wab (Fig. 1I), 1 wk later than in the control line (Fig. 1C). At 5 wab, when inflorescence arrest is already observed in the *ap2-170* mutant, the GUS signal was clearly visible in the mutant (Fig. 1J), in a similar pattern to that observed in the control line at 4 wab (Fig. 1D). This result suggested that the delayed meristem arrest observed in the *ap2-170* mutant could be associated with a delayed activation of *HB21* in the shoot apex.

In the *ful-2* mutant, the inflorescence meristem never experiences a complete arrest (Merelo et al. 2022), partly due to the derepression of several members of the *AP2* family, including *AP2* (Balanzà et al. 2018). In the *ful* mutant, the *proHB21:GUS* reporter activity was never detected during the entire flowering period (Fig. 1, K to N), and only a weak GUS signal could be observed several weeks (11 wab) after the onset of the arrest in wild-type plants, when the *ful* plants showed conspicuous signs of overall senescence (Fig. 1O).

**Figure 2.**
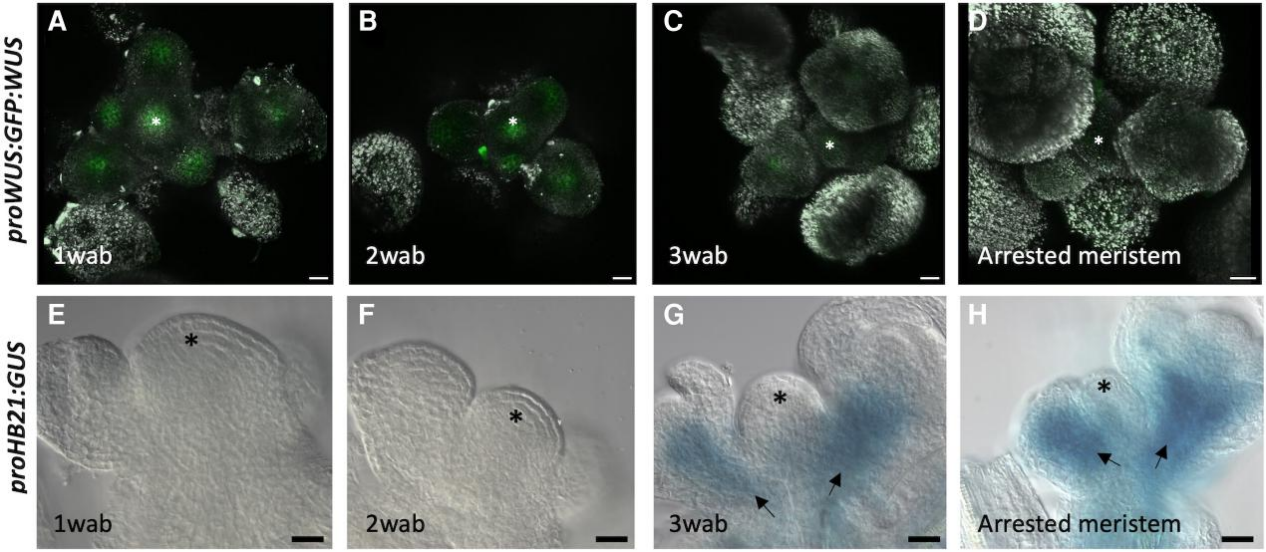
WUS accumulation negatively correlates with HB21 activation. The double reporter line *proWUS:GFP:WUS*-*proHB21:GUS* was analyzed. The same confocal imaged apexes were sequentially processed for GUS analysis. **A to D)** Expression of *proWUS:GFP:WUS* (green) 1 wab **A)**, 2 wab **B)**, 3 wab **C)**, and 4 wab (arrested; **D**). **E to H)** Histochemical detection of GUS activity driven by the *HB21* promoter at 1 wab **E)**, 2 wab **F)**, 3 wab **G)**, and arrested **H)**. At 3 wab, WUS level declines **C)** coinciding with *HB21* upregulation **G)**, and in arrested meristems, WUS protein is no longer detected while *HB21* promoter activity is at its highest **D, H)**. Arrowheads point to the floral buds. Asterisk marks the SAM. Bars represent 20 *µ*m.

The inflorescence meristem arrest is associated with a decline in *WUS* expression in the SAM, where it is no longer detected at the arrested stage (Balanzà et al. 2018; Merelo et al. 2022). To determine precisely when *HB21* accumulates in the shoot apex, we performed a simultaneous analysis of *WUS* activity and *HB21* expression during inflorescence development by introducing a *proWUS:GFP:WUS* reporter into the *proHB21:GUS* line. Individual apexes were sequentially processed for confocal detection of GFP:WUS and GUS analysis. As previously described, the GFP:WUS was detected at uniform levels until 3 wab, when it declined and eventually disappeared in arrested meristems, at 4 wab (Fig. 2, A to D). The decline in WUS accumulation at 3 wab coincided with the onset of *HB21* expression, and the WUS switched off a week later with the strong upregulation of *HB21* promoter activity (Fig. 2, E to H). The negative correlation of *WUS* and *HB21* supports again the idea that *HB21* initiates its expression when the SAM enters the low proliferative phase that leads to flowering termination, reaching its maximum level when the inflorescence meristem arrests. We also analyzed the signal of the *proHB21:GUS* reporter in the inflorescence apex after meristem reactivation by fruit removal (Hensel et al 1994). Once the meristem starts to produce new flowers, no GUS signal was detected in the apex (Supplementary Fig. S1) reinforcing the idea that *HB21* expression associates with inflorescence arrest.

### *HB21* induction promotes inflorescence arrest

**Figure 3.**
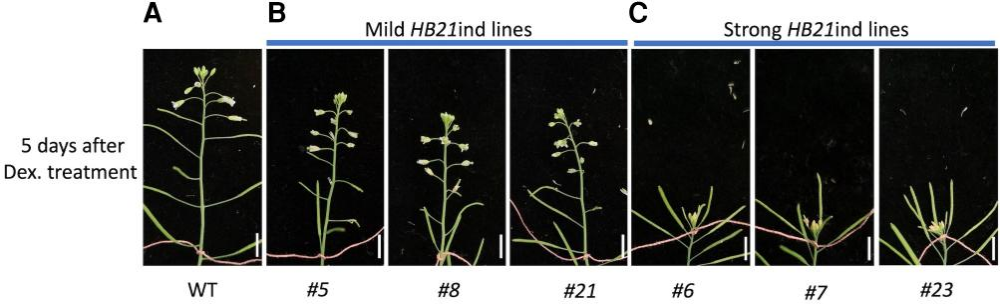
*HB21* induction forces flower and inflorescence arrest. Effect of the induction of *HB21* in proliferating inflorescences of wild-type plants (WT) 5 d after Dex treatment. **A)** WT plant treated with Dex, showing normal development. **B)** Representative WT *HB21*ind T1 lines showing mild phenotypes after Dex treatment, where flower development is arrested. **C)** Representative WT *HB21*ind T1 lines showing strong phenotypes after Dex treatment, where arrested inflorescence growth and floral bud senescence are observed. Pink ribbon marks point of Dex treatment. Bars represent 1 cm.

The negative correlation of inflorescence meristem activity and *HB21* expression suggested that the HB21 protein could be participating in the inflorescence arrest at the end of the flowering period. To test this hypothesis, we generated an inducible line of *HB21* (*pro35S:Lh-GR»HB21*, which we named as *HB21*ind; Moore et al. 2006). Selected T1 plants that expressed clearly the *HB21* gene were treated with dexamethasone (Dex), applying 1 drop to the main inflorescence apex at 2 wab, when the meristem is fully active, and checked 5 d after treatment. We identified 3 different categories of lines according to the effect of the Dex treatment in the inflorescence development: weak (or no response; 33.33% of lines), mild (23.33% of lines), and strong (43.33% of lines) in terms of inflorescence development. Treated weak lines were identical to the wild-type plants (Fig. 3A), where the inflorescence continued with a normal development, opening new flowers associated to stem elongation. Treated mild lines showed the developmental block of unpollinated floral buds (Fig. 3B), one of the landmark events that occurs during the end of flowering process (Walker et al. 2023). However, the induced mild lines did not show a visible effect in SAM activity, which stayed active producing new floral buds. In the other hand, strong lines showed a clear response to treatment on the whole meristem apex, inducing floral bud senescence and blocking inflorescence elongation, mimicking the inflorescence arrest (Fig. 3C).

To confirm the observed responses, 2 independent lines, 1 for the mild response group (line #21) and other for the strong response group (line #7), were selected and tested again in the T3 generation. We repeated the treatment performed in T1 plants, but now using as a control the mock-treated plants, obtaining the same phenotypes observed in T1 plants (Supplementary Fig. S2). Then, the results obtained with the *HB21*ind lines suggested that *HB21* is able to regulate the inflorescence development when expressed locally in the inflorescence apex and is sufficient to induce the arrest of the developing structures in the shoot apex, probably, in a dose-dependent manner.

### HB21 controls meristem arrest redundantly with HB53 and HB40

**Figure 4.**
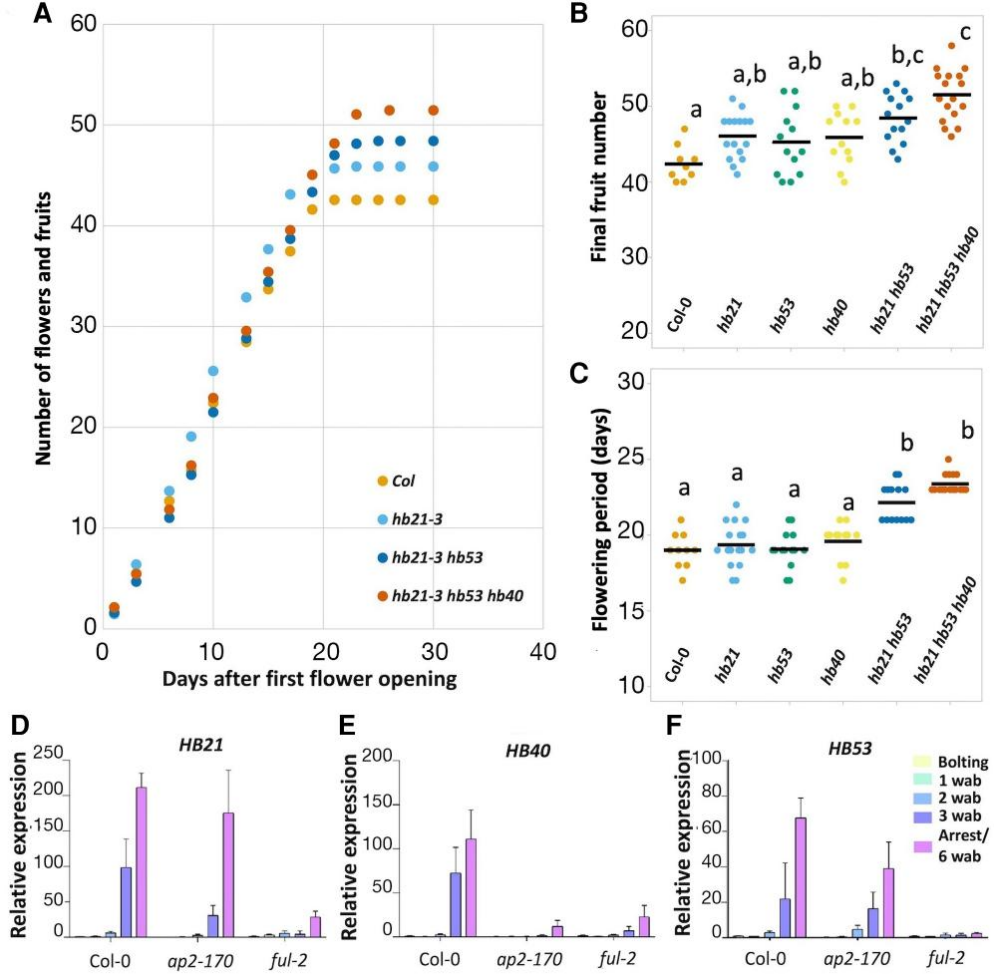
HB21, HB40, and HB53 act redundantly. **A)** Number of opened flowers and fruits produced over time by the main inflorescence of Col-0, *hb21*, *hb53*, *hb40*, *hb21 hb50*, and *hb21 hb53 hb40* plants. **B)** Total number of fruits produced in the main inflorescence of Col-0, *hb21*, *hb53*, *hb40*, *hb21 hb53*, and *hb21 hb53 hb40* plants. The final number of fruits increases gradually with the order of *hb* mutant combinations. **C)** Duration of flowering in the main inflorescence of Col-0, *hb21*, *hb53*, *hb40*, *hb21 hb53*, and *hb21 hb53 hb40* plants, quantified as the interval in days between the first to the last flower in anthesis observed. Double and triple *hb* mutants present extended flowering periods. **D to F)** Transcript levels of *HB21***D)**, *HB53***E)**, and *HB40***F)** at different time points of the flowering phase. All transcripts accumulate at the end of flowering in WT (arrest [4 wab]), are reduced in *ap2-170* mutants (4 wab), and almost not detected in *ful-2* mutants (6 wab). Dots in **A)** represent the average of at least 10 plants. Bars in **B, C)** represent the mean of each experiment, and each dot represents the value for 1 individual plant. A Kruskal–Wallis test followed by a Mann–Whitney *U* test was performed to assess statistical differences represented by lowercase letters **B, C)** (*P* < 0.05). The average of 3 biological replicates with the Sd as error bars is represented in **D to F)**.

Transcripts of *HB21* accumulate at the end of flowering, and its induction in the inflorescence apex is able to arrest inflorescence development. In agreement with this, it was previously proposed that *HB21* could modulate the end of flowering promoting inflorescence arrest based on the observation of *hb21-2* mutants (SAIL_790_D09.v1), which produced more flowers before inflorescence arrest than the control plants (Martínez-Fernández et al. 2020). To further characterize the role of *HB21* at the end of flowering, we decided to check an additional allele, *hb21-1* (WiscDsLox468G4), previously described for its role in bud dormancy (González-Grandío et al. 2017). Both mutations are caused by the insertion of a T-DNA in the third exon of the gene (Supplementary Fig. S3A), in very close positions (*hb21-1* at position 1404 and *hb21-2* at position 1487 from the ATG codon) at the 3′ end of the gene. Surprisingly, these 2 *hb21* mutant alleles showed different phenotypes related with the end of flowering. Wild-type plants produced 42.75 ± 2.52 fruits, while the *hb21-1* mutant produced 41.07 ± 2.21 in contrast with the 56.82 ± 9.46 fruits produced by the *hb21-2* mutant (Supplementary Fig. S3B). Because these alleles were likely not null, and to discard other putative second-site modifiers that could explain the disparity in phenotype of both mutant lines, we decided to generate additional mutant alleles by CRISPR/Cas9 genome editing. We selected a third allele for further characterization, *hb21-3*, with a deletion of 244 bp in the second exon of the gene that comprised the entire HD domain and likely caused the complete loss of function of the gene. The *hb21-3* plants did not show any apparent defects in plant architecture or organ development (Supplementary Fig. S4). When we compared the number of flowers produced before meristem arrest in the *hb21-3* mutant and the wild-type plants, a small but not significant difference was observed, producing 46.06 ± 2.95 and 42.44 ± 2.95 flowers, respectively (Fig. 4, A and B). This analysis indicated that the absence of *HB21* did not affect the number of flowers produced before arrest. The absence of phenotype in *hb21-3*, a null allele, compared with the increase in flower production observed in *hb21-2* (30% increase), suggested that the *hb21-2* is not a real loss-of-function mutant, or that this mutant line could carry an additional uncharacterized genetic lesion responsible for the observed phenotype.

Preliminary works with *HB21* indicated that this gene works redundantly with 2 additional genes, *HB40* and *HB53*, in the control of bud dormancy (González-Grandío et al 2017). *HB40* and *HB53* also accumulate at the end of flowering according to previously published transcriptomic data (Wuest et al. 2016), and *HB53* is repressed by *AP2* induction in the inflorescence similarly to *HB21* (Martínez-Fernández et al. 2020). To assess whether *HB21* could also work redundantly with *HB40* and *HB53* in the control of the end of flowering, we quantified the transcript accumulation of *HB21*, *HB53*, and *HB40* during the reproductive phase in the inflorescence apex by reverse transcription quantitative PCR (RT-qPCR). For this purpose, we collected dissected inflorescence apexes from wild-type plants and *ap2-170* and *ful-2* mutants at the same time points used in the *proHB21::GUS* analysis. The *HB21* transcript levels showed a clear increase in wild-type plants at 3 wab, reaching its maximum level in apexes of 4 wab when meristem arrest occurs (Fig. 4D). In the *ap2-170* mutant, the temporal pattern of *HB21* expression was similar to the observed in the wild-type plants, but the expression levels at 3 and 4 wab were significantly lower than in the control (Fig. 4D). Finally, the *HB21* expression in the *ful-2* mutant was very low during all the inflorescence development, being only slightly upregulated at 6 wab (Fig. 4D). The changes in the expression levels detected by RT-qPCR were in agreement with the GUS analysis reported for the *proHB21:GUS* line.

As observed for the *HB21* expression, *HB40* and *HB53* in the wild-type plants started to accumulate in the inflorescence apex 3 wab, reaching the highest expression level in arrested meristems (Fig. 4, E and F). The expression of *HB40* in the *ap2-170* mutant, where inflorescence arrest is delayed, and the *ful-2* mutant, where meristem arrest never happens, was low in all time points assessed, accumulating slightly in the arrested *ap2-170* apexes (4 wab) or in the apexes of *ful-2* mutants at 6 wab (Fig. 4E). The expression of *HB53* in the *ap2-170* mutant was similar to the expression observed in the wild-type plants but was not upregulated at the same extent at the moment of meristem arrest (Fig. 4F). The levels of *HB53* in the *ful-2* mutant were always low (Fig. 4F). Our analysis confirmed that *HB40* and *HB53* accumulated at high levels at the end of flowering as *HB21* did, and that their regulatory interactions with *AP2* and *FUL* were likely similar as well. Altogether, this supports the idea that the 3 *HB* genes might also act redundantly in the control of proliferative arrest at the end of the flowering phase.

To test this hypothesis, we characterized the *hb40-1* and *hb53-1* (thereafter *hb40* and *hb53*) single mutants as well as the *hb21 hb53* and *hb21 hb53 hb40* double and triple mutants. The single mutants *hb53* and *hb40* produced in average a small increase in the final number of fruits (45.23 ± 4.32 and 45.83 ± 3.46) with respect to the wild-type control plants (42.44 ± 2.35; Fig. 4B; Supplementary Fig. S5, A and B), but none of them were statistically significant. The double mutant *hb21 hb53* produced a significant increase in the final number of fruits (48.43 ± 3.20) with respect to the wild-type plants, but not with respect to the *hb* single mutants (Fig. 4, A and B). Finally, the triple *hb21 hb53 hb40* presented the stronger effect in the number of fruits produced by the inflorescence with respect to the wild-type plants as well as to the *hb* single mutants, with a production of 51.47 ± 3.39 fruits.

The *HB* genes are expressed at the end of the flowering period, from the stage when the meristem declines in proliferative capacity until the arrest. Thus, *HB* genes should exert their function at this developmental stage. In agreement with this, the different mutants characterized did not affect meristem activity during the first weeks of flower production, measured as the rate of flower opening (Fig. 4A). We calculated then the flowering period of the inflorescence (days between first and last opened flower), and no differences were observed between the wild-type plants and all the single mutants (Fig. 4, A and C). In contrast, both the double and triple mutants showed a clear extension in the flowering period with respect to the wild-type plants. Last opened flower in the wild type occurred at 19 ± 1.15 d after the opening of the first one (Fig. 4, A and C). In the *hb21 hb53* and *hb21 hb53 hb40* mutants, the last flower opened at 21.71 ± 0.99 and 21.81 ± 0.98 d, respectively (Fig. 4, A and C). Our results indicated that the *hb* mutants increased the final number of fruits produced through an extension of the flowering period.

**Figure 5.**
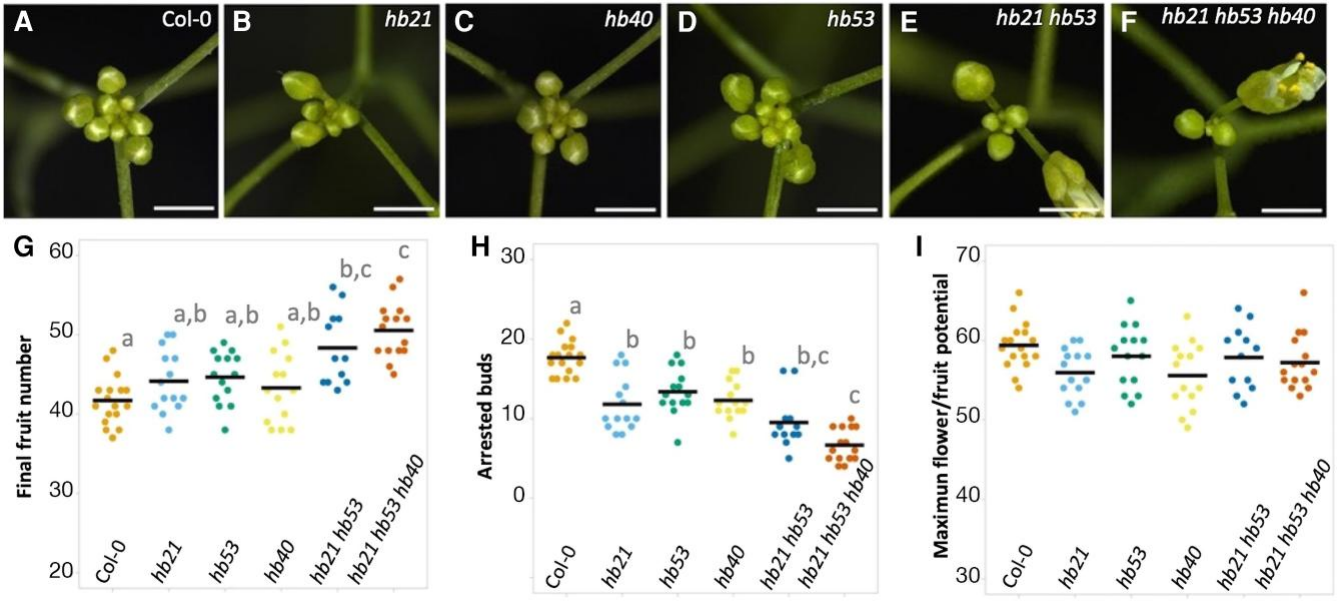
*HB* genes promote bud arrest. **A to F)** Representative bud cluster phenotypes of Col-0 plants **A)**, single **B to D)**, double **E)**, and triple **F)***hb* mutants at the end of flowering. **G)** Final number of fruits in the main inflorescence. The number of fruits developed in the main inflorescence increases gradually in the single, double, and triple *hb* mutants. **H)** Number of arrested buds in the final cluster of the main inflorescence. Buds present in the final cluster decrease gradually in the single, double, and triple *hb* mutants. **I)** MFP. No differences are observed in the MFP of the different genotypes analyzed. Bars in **A to F)** represent 1 mm. Bars in **G to I)** represent the mean of each experiment, and each dot represents the value for 1 individual plant. A Kruskal–Wallis test followed by a Mann–Whitney *U* test was performed to assess statistical differences (*P* < 0.05) represented by lowercase letters.

Interestingly, when we inspected the cluster of arrested flowers formed at the end of flowering in the *hb* mutants, we realized that they differed in size. While the wild type and single mutants showed similar sizes, the double and triple mutants developed smaller clusters with fewer buds (Fig. 5, A to F). Then, we decided to perform an experiment comparing the final number of fruits produced and the number of floral buds present in the arrested bud cluster of the wild type and the *hb* mutants. The fruits produced by the different plants (Fig. 5G) were similar to those in the previous experiment (Fig. 4B) with the *hb21 hb53 hb40* mutant forming the higher number of fruits (Fig. 5G). When we counted the number of buds present in the final inflorescence cluster, we obtained opposite results. The bud cluster in the wild-type plants contained an average of 17.64 ± 2.12 arrested buds, while the single mutants *hb21*, *hb53*, and *hb40* contained 11.78 ± 3.46, 13.36 ± 2.84, and 12.29 ± 2.30, respectively (Fig. 5H). The difference was more evident in the double and triple mutants *hb21 hb53* (9.5 ± 3.32) and *hb21 hb53 hb40* (6.67 ± 2.06 arrested buds; Fig. 5H). If we consider the total number of primordia produced by the inflorescence meristem (maximum flower/fruit potential [MFP]), we observed no differences between the control and the different mutants characterized (Fig. 5I).

Altogether, our results indicated that the *HB21*, *HB53*, and *HB40* genes regulate the final number of fruits produced in the inflorescence redundantly, promoting floral bud arrest at the end of flowering without affecting meristem activity, since the increase in the number of developed fruits observed in the triple *hb21 hb53 hb40* mutant could be explained by the delayed floral arrest already present in the inflorescence apex.

### HB21 controls inflorescence arrest controlling ABA biosynthesis and response

Our results indicated that *HB* genes participate in the control of the end of flowering. To obtain more clues on the mechanism and the regulatory networks acting downstream these genes, we decided to perform a whole transcriptomic analysis. The inducible *HB21* line was used to overcome the redundancy with *HB53* and *HB40*. We treated inflorescence apexes of 2 wab *HB21* inducible lines (active meristems with low endogenous expression of *HB21*) with Dex or mock. Then, 6 h posttreatment, we collected inflorescence apexes. Three independent biological replicates for each treatment were used for RNA sequencing. Transcripts with a log_2_ fold change (FC) > 1 and <−1 and a *P*-adjusted value < 0.05 were considered as differentially expressed genes (DEGs) and selected for further analysis. We obtained 1,143 DEGs, 471 activated and 672 repressed by *HB21* induction (Supplementary Table S1).

**Figure 6.**
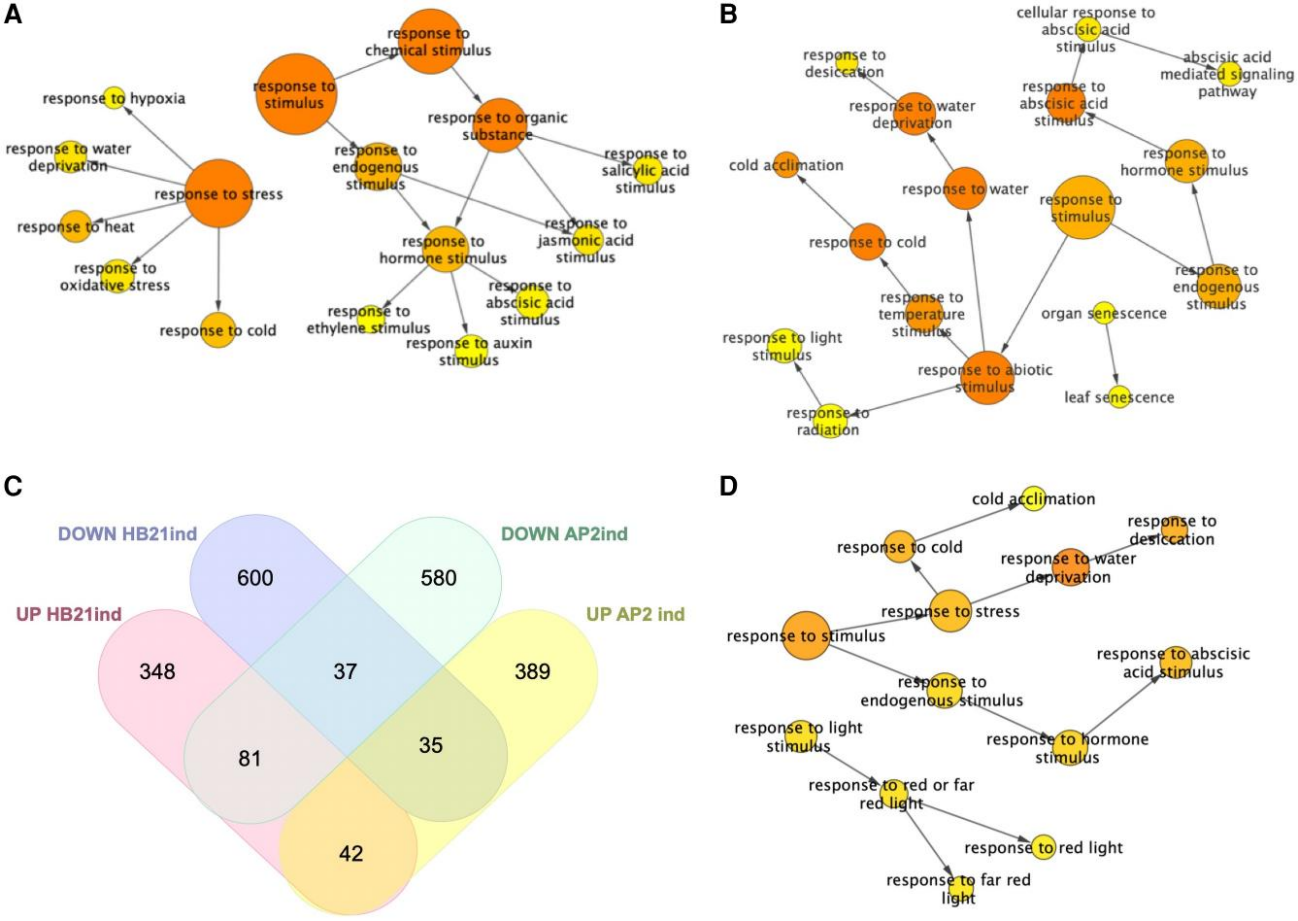
Functional enrichment analysis with overrepresented GO biological process categories. **A, B)** The analysis was performed for total DEG, upregulated **A)** and downregulated **B)** by *HB21* induction. **C, D)** Comparison of HB21 induction and AP2 induction (Martínez-Fernández et al. 2020) DEG. The Venn diagram showed DEG shared between *HB21* induction and *AP2* induction **C)**. Go analysis of the 81 DEGs upregulated by *HB21* and downregulated by *AP2***D)**. All analyses share response to cold, water deprivation, and ABA stimulus **A, B, D)**. Circle size is proportional to gene numbers, and the color of each circle represents the enriched *P*-value (hypergeometric test) for the GO term label on that circle, with orange representing the highest enrichment and yellow the lowest enrichment above the cutoff (Benjamin and Hochberg false discovery rate corrected 0.05). Some categories were removed, and the distance between nodes was arranged manually to optimize readability. The figure and statistical analysis were generated using the BiNGO software.

We conducted a Gene Ontology (GO) analysis using the BiNGO tool (Maere et al. 2005) implemented for Cytoscape (Shannon et al. 2003), focusing in the enriched terms in the category Biological Process. For the downregulated DEGs, we found 69 categories overrepresented, including the response to multiple stimuli and stress (Supplementary Table S2). Within the “response to stimulus” category, the response to hormones like jasmonic acid (12 genes), salicylic acid (11genes), ABA (16 genes), auxin (15 genes), and ethylene (9 genes) stood out (Fig. 6A). The “response to stress” category included response to hypoxia (4 genes), to water deprivation (14 genes), to heat (14 genes), to cold (19 genes), and to oxidative stress (16 genes; Fig. 6A). For the upregulated DEGs, we found 53 categories overrepresented that included similar categories to the observed in the downregulated group (Supplementary Table S3), highlighting the response to ABA (25 genes), water deprivation (31 genes), and cold (21 genes; Fig. 6B). In addition, the response to light (17 genes) was also overrepresented together with the categories “leaf and organ senescence” (4 genes; Fig. 6B). This analysis indicated that the induction of *HB21* was able to modulate the response to multiple stimuli, both endogenous and exogenous. It has been described that meristem arrest at the end of flowering is associated with an increased ABA response and resembles a state of bud dormancy (Wuest et al. 2016). The transcription factor AP2 represses proliferative arrest, at least in part, by the repression of the ABA response. As AP2 is a direct negative regulator of *HB21*, we decided to analyze which part of the role of AP2 in proliferative arrest was likely mediated by *HB21*. Thus, we compared the DEGs responding to the induction of *AP2* (Martínez-Fernández et al. 2020) with the DEGs responding to *HB21* induction. AP2 and HB21 exert opposite effects on meristem arrest: AP2 promotes inflorescence activity while HB21 promotes inflorescence arrest and senescence. Thus, we focused on genes that showed an opposite behavior in both experiments. We found that a total of 116 genes showed this pattern, 81 were upregulated by HB21 and downregulated by AP2, and 35 were downregulated by HB21 and upregulated by AP2 (Fig. 6C). An additional GO analysis with these 2 subsets of genes (Supplementary Table S4) revealed that the group of genes upregulated by HB21 and downregulated by AP2 was also enriched in the categories of response to stress, including the response to cold (8 genes) and the response to water deprivation (13 genes), and the response to endogenous stimulus, standing out the response to ABA (8 genes; Fig. 6D). In the complementary group, only the categories sulfur metabolic process (4 genes) and sulfate assimilation (3 genes) stood out.

Our analysis suggested that *HB21* could mediate the ABA responses that appeared repressed by AP2. Once HB21 accumulates in the inflorescence apex, it could trigger the ABA response. In agreement with this hypothesis, we identified in the list of upregulated DEGs 2 key genes in the biosynthetic ABA pathway, *NCED3* and *NCED4* (Iuchi et al. 2001; Tan et al. 2003), with log_2_ FC values of 4.35 and 1.77, respectively (Supplementary Table S1). Thus, we hypothesized that upregulation of *HB21* induces ABA biosynthesis and the subsequent ABA response, and that this upregulation could mediate the floral arrest at the end of flowering.

**Figure 7.**
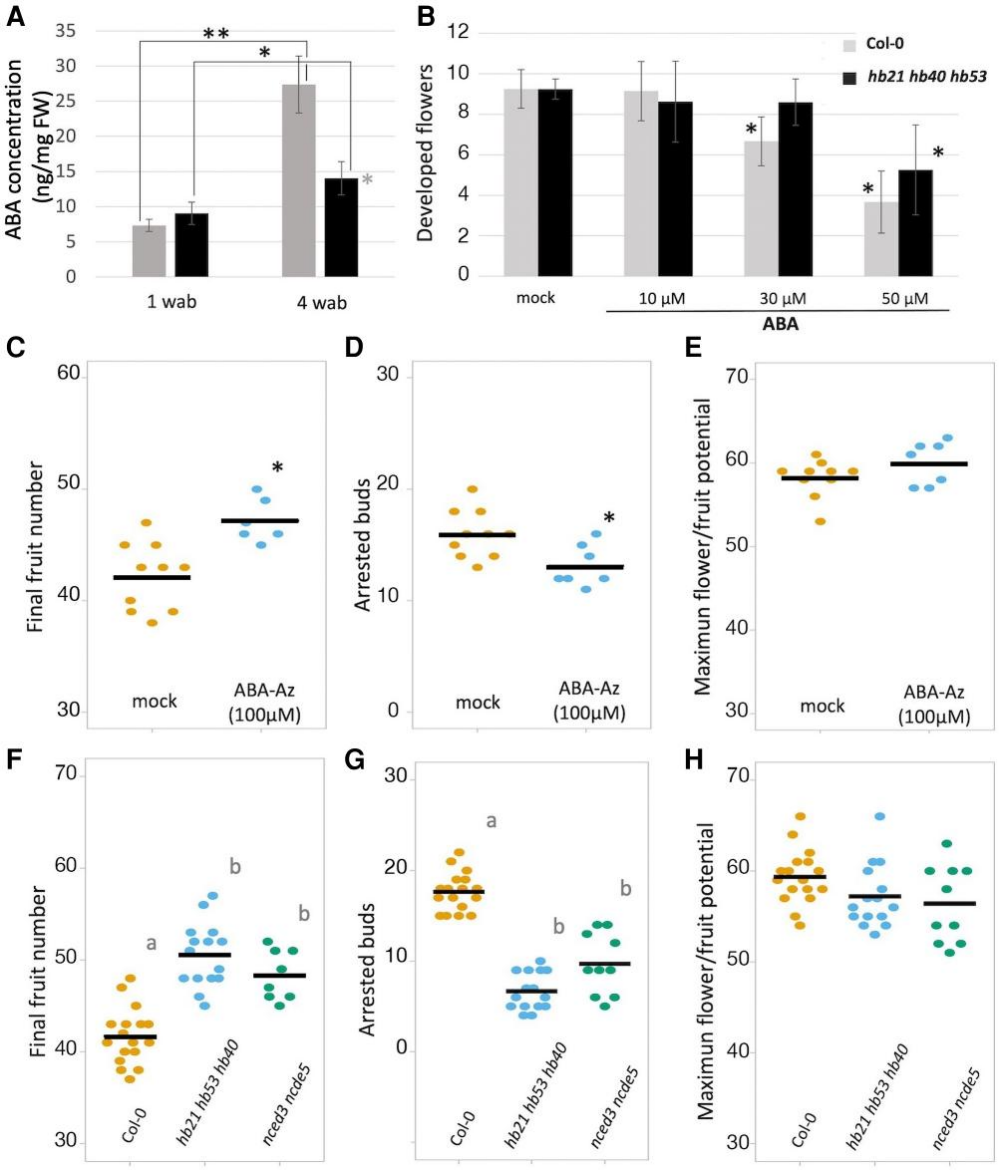
ABA controls the developmental block of floral buds at the end of flowering. **A)** ABA levels at the end of flowering in inflorescence apexes. ABA accumulation is observed in wild-type Col-0 apexes at the end of flowering, while triple *hb* mutants accumulate lower levels. **B)** Effect of ABA treatment on active inflorescence apexes. ABA blocks flower development in both wild-type and triple *hb* mutant plants in a concentration-dependent form. The triple *hb* mutant requires higher levels of ABA to block flower development. **C, D)** Effect of ABA-Az ABA antagonist treatment on inflorescence apexes. Treatment increases the final number of fruits developed in the main inflorescence **C)**, decreasing the number of arrested buds present in the final cluster **D)**. The MFP is not affected by ABA-Az treatment **E)**. **F to H)** Characterization of an ABA biosynthetic-deficient mutant (*nced2nced5*). *nced2nced5* mutant phenotype resembles the triple *hb* mutant and ABA-Az-treated plants. *nced2nced5* mutant increases the final number of fruits developed in the main inflorescence **F)**, decreasing the number of arrested buds present in the final cluster **G)** without changes in the MFP **H)**. In **A)**, black asterisk indicates significant differences (*P* < 0.05) from the young inflorescences while gray asterisk indicates significant differences (*P* < 0.05) at the same time point (Student's *t* test). Each block represents the average of 3 samples, and errors bars correspond to the Sd. FW means fresh weight. In **B to D)**, asterisk indicates significant differences (*P* < 0.05) from the mock-treated apexes according to Student's *t* test. In **B)**, each block represents the average of at least 7 plants, and error bars correspond to the Sd. Bars in **C to H)** represent the mean of each experiment, and each dot represents the value for 1 individual plant. A Kruskal–Wallis test followed by a Mann–Whitney *U* test was performed to assess statistical differences (*P* < 0.05) represented by lowercase letters in **F and G)**.

ABA levels in the inflorescence apexes were increased upon *HB21* induction after 6 and 24 h of DEX treatment (Supplementary Fig. S6), indicating that HB21 can promote ABA accumulation. To assess if ABA levels are also elevated in physiological conditions at the end of flowering, we quantified ABA levels in proliferative inflorescences 1 wab, and in apexes at 4 wab, close to the inflorescence arrest, in both wild type and *hb21 hb40 hb53* mutant. In wild-type plants, ABA levels increased significantly at 4 wab with respect to early stages (1 wab; Fig. 7A). When we compared the triple *hb21 hb40 hb53* mutant with the wild-type plants, no differences were observed at 1 wab, in proliferative apexes. However, at 4 wab, when *HB21/40/3* levels are high in the inflorescence apex, ABA levels were significantly lower in the triple mutant than in the wild type (Fig. 7A). These results suggested that the ABA increase in the inflorescence apex at the end of flowering was dependent on the *HB21/40/53* genes, and that this ABA accumulation could mediate the floral arrest associated to the end of the flowering phase.

To further test this hypothesis, we decided to check the effect of exogenous ABA on inflorescence development by applying local ABA treatments to whole inflorescence apexes of wild-type and the triple *hb21 hb53 hb40* mutant plants. We applied a drop of a 10, 30, or 50 *µ*M ABA solution to 2 wab inflorescence apexes (proliferative) during 3 consecutive days and scored the phenotypes 2 d after the last treatment. The ABA treatment affected both the wild type and the triple mutant similarly, producing an effect in the inflorescence apex resembling the typical morphology of apexes at the end of flowering, with a reduction of stem elongation and the developmental block of the already formed floral buds (Fig. 7B; Supplementary Fig. S7). ABA treatments had a stronger effect on wild-type plants (Supplementary Fig. S7), affecting flower development progression from 30 *µ*M, while in the triple mutant, a 50 *µ*M ABA concentration was necessary to obtain a significative effect (Fig. 7B).

Conversely, to unequivocally address a role of ABA in the control of inflorescence arrest, we also treated plant inflorescences with an ABA receptor antagonist (ABA-Az). This compound binds to ABA receptors preventing their interaction with ABA coreceptors impairing therefore ABA signaling (Supplementary Methods S1 and Fig. S8). We applied a drop of a 100 *µ*M solution of ABA-Az every 3 d on the inflorescence apex until inflorescence arrest took place. In agreement with our hypothesis, the decrease in ABA perception caused by the antagonist treatment delayed the floral arrest. Mock-treated plants produced an average of 42.20 ± 3.05 fruits, with final clusters formed by 16.00 ± 2.16 buds, while ABA-Az-treated plants produced 47.12 ± 1.94 fruits and 13.14 ± 1.86 buds (Fig. 7, C and D). Interestingly, the treatments did not affect the maximum floral potential of both the mock- and ABA-Az-treated plants, being 58.20 ± 2.25 and 60.00 ± 2.58, respectively (Fig. 7E), strongly suggesting that ABA did not control inflorescence meristem activity. Finally, we also characterized the phenotype of a *nced3 nced5* double mutant, where ABA synthesis is impaired (Iuchi et al. 2001; Tan et al. 2003; Frey et al. 2012). The *nced3 nced5* mutant produced in average a clear increase in the final number of fruits (48.37 ± 2.72) with respect to the numbers observed in the wild-type control plants (41.70 ± 3.05) but no differences with respect to the triple *hb21 hb53 hb40* mutant (50.53 ± 3.48; Fig. 7F). When we analyzed the bud cluster of these plants, we observed that while the wild-type plants contained an average of 17.64 ± 2.12 arrested buds, the *nced3 nced5* mutant contained a clear lower number, 9.70 ± 3.40 (Fig. 7G). Again, the number of arrested buds in the *nced3 nced5* mutant was similar to the obtained in the *hb21 hb53 hb40* mutant (6.67 ± 2.06; Fig. 7G). Finally, when the total number of floral buds produced by the inflorescence meristem (MFP) was analyzed, no differences between the control, the *nced3 nced5*, and the *hb21 hb53 hb40* mutants were observed (59.35 ± 2.99, 56.40 ± 4.27, and 57.20 ± 5.49 buds, respectively; Fig. 7H). As expected, the *nced3 nced5* mutant, unable to accumulate high ABA levels, presented a phenotype similar to the double and triple *hb* mutants, and in line with the results obtained with the ABA receptor antagonist treatments, increasing the number of fruits produced through a reduction of the final bud cluster. Then, our results suggested that high ABA levels were able to trigger the floral arrest at the end of flowering.

## Discussion

The mechanisms that control the end of flowering in monocarpic plants have only started to be elucidated in the last few years in Arabidopsis. The inflorescence meristem proliferative arrest associated with the end of flowering is under genetic control, where the AP2 transcription factor acts as a key negative regulator of the process (Balanzà et al. 2018; Martínez-Fernández et al. 2020). The inflorescence arrest is influenced by environmental factors such as light quality and photoperiod or temperature (Martínez-Fernández et al. 2020; González-Suárez et al. 2023), as well as by endogenous factors such as age, auxin, and CK dynamics in the meristem (Martínez-Fernández et al. 2020; Ware et al. 2020; Goetz et al. 2021; Merelo et al. 2022; Walker et al. 2023). A putative role of ABA signaling in meristem arrest was suggested by transcriptomic profiling of inflorescence meristems at different stages throughout the flowering period (Wuest et al. 2016; Martínez-Fernández et al. 2020). Here, we provide evidence that places *HB21*, a direct target of AP2, together with *HB53* and *HB40*, as a promoter of the ABA responses associated with the end of flowering through the activation of ABA biosynthesis, confirming as well a role for *HB* genes and ABA in the control of the inflorescence arrest.

*HB21*, *HB53*, and *HB40* have been associated to the gene regulatory network that controls the dormancy of axillary buds in Arabidopsis. The 3 genes are activated by the TCP factor BRC1 in the axillary meristems (González-Grandío et al. 2013, 2017). This activation occurs very early in development and, together with other factors controlled by BRC1 (van Es et al. 2024), restricts the outgrowth of these meristems. This outgrowth block mediated by BRC1 is partially dependent on ABA (van Es et al. 2024) and the action of the *HB* genes characterized here (González-Grandío et al. 2017). We have shown that *HB21*, *HB53*, and *HB40* are expressed in the inflorescence apex when the low proliferative phase of the meristem starts (Merelo et al. 2022), increasing its levels until the inflorescence arrest. Our results indicate that *HB21*, *HB53*, and *HB40* control inflorescence arrest activating ABA biosynthesis and response as they do in axillary meristems, mediating the developmental block of the last floral buds produced by the inflorescence. Altogether, our work uncovers a physiological role for the hormone ABA. *HB* genes promote ABA biosynthesis and accumulation, probably through the induction of *NCED3* and *4*. This is supported by the observation that the triple *hb* mutant does not accumulate ABA levels as high as the wild-type plants at the end of flowering. In agreement with this, we have shown that a deficient mutant in ABA biosynthesis mimics the triple *hb21 hb40 hb53* mutant phenotype, pointing out that ABA is involved in the floral arrest at the end of flowering. The ABA role in the developmental block of floral buds is also confirmed by local ABA treatments in the inflorescence apex showing that ABA is able to arrest flower development in active inflorescences (2 wab). In addition, *HB21* induction produces a fast increase in ABA levels that is translated in the later developmental block of floral buds, indicating again that ABA could mediate flower development arrest. These results were confirmed using an ABA receptor antagonist, which prevented the floral arrest, mimicking the triple *hb* and the ABA-deficient mutant phenotypes. Thus, we propose ABA as an important determinant in the control of inflorescence arrest. Supporting this hypothesis, in drought conditions, where ABA levels are elevated, an early and transitory inflorescence arrest has been described (Su et al. 2013), mainly affecting flower development.

Axillary bud outgrowth requires, between others, the increase in CK levels that counteract the ABA repressive action, in part by the repression of the *BRC1* expression and consequently *HB* gene expression (Shimizu-Sato et al. 2009; Müller and Leyser 2011; Sreenivasulu et al. 2012; Reddy et al. 2013; Yao and Finlayson 2015; González-Grandío et al. 2017; Tarancón et al. 2017; Schneider et al. 2019). Similarly, inflorescence growth and flower production also require CK, which are essential for meristem maintenance, promoting cell division and *WUS* expression (Bartrina et al. 2011; Schaller et al. 2014; Bartrina et al. 2017; Meng et al. 2017; Merelo et al. 2022; Walker et al. 2023). We have shown that *HB* expression in the apex correlates with the *WUS* expression and CK response decline (Merelo et al. 2022). It has been proposed that elevated ABA levels, as induced by drought, could inhibit the biosynthesis of CK and therefore the CK responses (Abe et al. 1997; Vaseva et al. 2008). Then, the ABA accumulation activated by the *HB* gene expression could participate in the progressive repression of the CK response observed at the end of flowering (Merelo et al. 2022). Likewise, the lower CK response observed in the last stages of inflorescence development could cause ABA hypersensitivity (Nishiyama et al. 2011), enhancing the ABA response. We have shown that ABA is present in young inflorescences, when the stronger CK response has been described (Merelo et al. 2022), but its level increase drastically close to the end of flowering in an HB-dependent manner, correlating with a weak CK response. That means that both hormones are acting simultaneously along inflorescence development, but at different ratios. Altogether, we could hypothesize that the balance between these hormones, ABA and CK, would play a determinant role in the control of inflorescence activity.

The end of flowering is characterized by 2 main events: floral arrest and apex arrest (Walker et al. 2023). Recently, based on macroscopic observations, it has been described that meristem arrest should occur before the floral arrest, quite early during inflorescence development (Walker et al. 2023). Our experiments show a negative correlation between WUS decline and *HB21* activation, suggesting that both the floral arrest and the inflorescence meristem arrest could be closely coupled. In our mild inducible line, the main phenotype observed was the developmental block of floral buds, without effects on stem elongation or inflorescence activity, supporting the idea that developmental block of floral buds and meristem arrest are 2 separate processes as proposed by Walker et al. (2023). Interestingly, the flowering periods of these 2 works (Walker et al. 2023; this work) differ considerably, suggesting that the 2 processes could be affected by specific growth conditions, as could be light quality. Interestingly, *HB* genes have been proposed to control bud dormancy in specific growth conditions as low red/far-red ratios or short-day conditions (González-Grandío et al. 2017). Thus, *HB* genes could connect both the floral arrest and the meristem arrest depending on specific environmental conditions, but further studies are necessary to clarify it.

Our results indicate that *HB21* is expressed locally in floral buds, and *hb* mutants apparently do not affect meristem activity, as observed in the triple *hb* mutant. In fact, MFP of both wild type and triple *hb* mutant are identical, although additional experiments should be performed in order to determine if *HB* genes could affect meristem structure or cell division rate. Once HB21 levels are high enough in floral buds, it could also contribute to meristem arrest in a noncell autonomous manner, well by the activation of a second factor and well by ABA transport to the adjacent tissues of the meristem (Kuromori et al. 2010). Additionally, we cannot discard that *HB* genes could be acting directly on the meristem, as the transcript of this gene has been detected at high levels at the meristem arrest stage by RNA-seq from microdissected samples of the SAM (Wuest et al. 2016).

In barley, the final number of spikelets is controlled by a developmentally programed process known as the preanthesis tip degeneration (PTD; Huang et al. 2023; Shanmugaraj et al. 2023). After the production of a certain number of spikelet primordia, PTD starts with the growth arrest of the inflorescence meristem dome, which is followed basipetally by the developmental block of floral primordia in the tip of the inflorescence. While this process could be reminiscent of the proliferative arrest of the inflorescent meristem described in monocarpic dicot plants, it is unclear whether these are homologous processes, given the profound morphological and ontogenetic differences between barley inflorescence and the Arabidopsis simple raceme. Here, we propose a role for HB21/40/53 and ABA in Arabidopsis inflorescence arrest that is almost identical to the recently proposed model in barley for PTD, where the HD-ZIP gene *GRASSY TILLERS* (*HvGT1*), a close paralog of *HB21/HB40/HB53*, is expressed specifically in the inflorescence apex at the end of inflorescence development (Huang et al. 2023; Shanmugaraj et al. 2023). Then, *HvGT1* triggers the expression of *HvNCED1* and ABA accumulation, which initiates inflorescence tip degeneration. Despite the wide phylogenetic distance between both species, the apparent conservation of the role of the *HB* genes in the control of the final number of flowers produced by the inflorescence meristem in both species suggests that the proposed mechanism could be present in many plants with indeterminate inflorescences and that PTD and inflorescence proliferative arrest are related processes.

Finally, this role of HB21, HB40, and HB53 controlling the floral arrest at the end of flowering could provide a biotechnological approach to boost yield, at least in some brassica crops. Thus, the selection of new lines with mutations in the described genes, as well as the use of ABA antagonists, could force the plants to develop the maximum flower potential of the inflorescences.

## Materials and methods

### Plant material and growth conditions

Arabidopsis seeds were stratified for 3 d at 4 °C after sowing. Plants were grown in cabinets at 21 °C under LD (16-h light/8-h dark) conditions, in a 2:1:1 (v/v/v) mixture of peat:perlite:vermiculite. All mutant plants and marker lines used in this study were in the Columbia background, except for *pWUS::GFP:WUS* that was in Landsberg *erecta*. Mutant alleles and transgenic lines have been previously described: *ful-2* (Ferrandiz et al. 2000), *ap2-170* (Balanzà et al. 2018), *hb21-1*, *hb40-1*, *hb53-1*, *proHB21::GUS* (González-Grandío et al. 2017), *hb21-2* (Martínez-Fernández et al. 2020), and *pWUS::GFP:WUS* (Yadav et al. 2011). The *35S::LhG4:GR»HB21* construct was generated by Gateway cloning of the *HB21* CDS into the pOpOn2.1 binary vector (Moore et al. 2006). The mutant combinations were generated by crossing. The *hb21-3* allele was created by CRISPR following Wang et al. (2015) using the web tool (http://crispr.hzau.edu.cn/cgi-bin/CRISPR2/CRISPR) to design the 2 RNAg to generate a deletion (Supplementary Table S5). The deletion was confirmed by PCR and sequencing. Primer sequences used are detailed in Supplementary Table S5. In all cases, Arabidopsis was transformed with *Agrobacterium tumefaciens* strain C58 pM090 using the floral dip protocol (Clough and Bent 1998), and both homozygous CRISPR lines and transgenic lines carrying a single transgene insertion were selected.

### GUS staining

For GUS histochemical detection, samples were treated for 20 min in ice-cold 9/1acetone/water (v/v) and then washed for 5 min with washing buffer (50 mM sodium phosphate [pH 7], 2 mM ferrocyanide, 2 mM ferricyanide, and 0.2% Triton X-100) and incubated O/N at 37 °C with staining buffer (washing buffer + 2 mM X-gluc). Following staining, plant material was fixed and cleared in chloral hydrate. Samples were mounted to be viewed under bright-field microscopy Leica DM5000.

### Confocal microscopy

Live imaging analyses were performed on a Stellaris 8 FALCON confocal microscope (Leica) using a water-dipping 40× objective. Reproductive shoot apices were imaged under water on MS medium plates and with the stem embedded in the MS medium. To allow a proper exposition of the shoot apex during live imaging, all flower buds were carefully removed with clean tweezers and a fine needle. GFP was imaged using a WLL (Supercon) laser emitting at the wavelength of 489 nm with a 22.65% intensity together with a 494 to 533 nm collection bandwidth (250 gain). Z stacks were acquired with a resolution of 8-bit depth, section spacing of 0.1 mm. More than 5 SAMs were observed.

### Dex treatment

Plants were grown in soil until 2 wab. The induction of 35S::LhG4:GR»HB21 in the shoot apex of transgenic plants was carried out by applying 1 drop (3 *μ*L) of a Dex solution (10 *µ*M Dex and 0.015% [v/v] Silwet L-77) or a control solution with an equivalent concentration of Silwet L-77 (mock) in the shoot apex. Plants were observed 5 d later. For the RNA-seq, inflorescence apices were harvested and dissected to eliminate older buds 6 h after induction, and 3 biological replicates were sampled, each containing about 16 inflorescence meristems.

### RT-qPCR

Inflorescence meristems were trimmed to remove all opened flowers. Three biological replicates were sampled, each containing 16 inflorescence meristems. RNA was extracted using the E.Z.N.A. Plant RNA Kit (Omega Bio-tek) and DNase treated with EZNA RNase-Free DNase I (Omega Bio-tek). RNA concentration and purity were verified using a NanoDrop Spectrophotometer ND-1000 (Thermo Scientific). cDNAs were synthesized from 800 ng of total RNA using random hexamers and SuperScript IV (Invitrogen). The RT-qPCR was performed in the QuantStudio 3 Real-Time PCR (Thermo Fisher) and used SyberGreen to monitor double-stranded DNA synthesis. The Ct value was obtained from an automatic threshold. Results were normalized to the expression of the TIP41 reference gene. The 2^−ΔCt^ was shown as relative expression level. Three technical replicates were performed for each biological sample, and the average of the 3 biological samples was represented in the figures. Primer sequences used are detailed in Supplementary Table S5.

### Fruit/flower and bud number quantification

For final fruit quantification, elongated fruits were quantified in the main inflorescence for at least 10 plants of each genotype after meristem arrest. For the accumulative number of flowers produced by the inflorescence, all floral nodes produced until the last flower in anthesis were counted each 2/3 d in the main shoot. Unhealthy plants were discarded. After inflorescence arrest, bud clusters were collected and dissected under a stereoscope counting all buds present. Experiments were replicated independently twice, obtaining comparable results, although only 1 experiment is represented in each figure.

### RNA-seq

RNA for RNA-seq was obtained with the RNeasy Plant Mini Kit (QUIAGEN), DNase included in the Kit. RNA integrity was determined according to RNA Integrity Number values using a Bioanalyzer Chip RNA 7500 series II (Agilent). The RNA-seq was performed by Novogene Company United, with 20M reads. For the bioinformatic analysis, reads were aligned to the reference genome of Arabidopsis available at the TAIR database (Lamesch et al. 2012) using TopHat (Trapnell et al. 2009) and Bowtie (Langmead et al. 2009) software. The abundance estimation of the transcripts was performed using the RSEM package (Li and Dewey 2011), and the differentially expressed transcripts (fragments per kilobase million value) were estimated using Cufflinks (Trapnell et al. 2010). The sequences from DEG were annotated through BLAST search against the TAIR database. DEGs were analyzed using the BiNGO tool (Maere et al. 2005) implemented for Cytoscape (Shannon et al. 2003), focusing in the enriched terms in the category Biological Process. The RNA-seq data discussed in this article have been deposited in the National Center for Biotechnology Information's Gene Expression Omnibus (Edgar et al. 2002) and are accessible through GEO Series accession number GSE249766 (https://www.ncbi.nlm.nih.gov/geo/query/acc.cgi?acc=GSE249766).

### ABA and ABA-Az treatments

For experiments on inflorescences, 2 wab, ABA/ABA-Az or a control treatment (MOCK) was applied. For this, 1 drop (3 to 5 *μ*L) of ABA solution (10/30/50 *µ*M, 0.015% Silwet L-77) or ABA-Az solution (100 *µ*M, 0.015% Silwet L-77) and the respective MOCK (0.015% Silwet L-77) was added to the inflorescence apex. For ABA, the treatment was repeated for 3 d, and the plants were observed 2 d later. For ABA-Az, the treatment was applied every 3 d until inflorescence arrest. For germination experiments, seeds were sown on 24-well plates containing MS/2 0.5% sucrose 1% agar media, including different concentrations of ABA and/or ABA-Az using 0.2% DMSO as control. After 3-d stratification at 4 °C, plates were incubated in a growth chamber under LD conditions (16-h light/8-h dark) at 24 °C/22 °C (day/night) for 3 d. Images were collected using a Leica macroscope. The experiments were performed 3 times.

### ABA-Az synthesis

Detailed procedure is described in Supplementary Methods S1.

### Protein purification and PP2C in vitro assays

Recombinant PYL1 and PYL10 ABA receptors and dNHAB1 protein phosphatase were produced and purified as described previously (Lozano-Juste et al. 2021). PP2C enzymatic assays were carried out using 4-MUP in the presence or absence of different concentrations of ABA and the antagonist ABA-Az using a 1:1 ratio receptor: phosphatase as reported (Okamoto and Cutler 2018). Assays were conducted in triplicate and repeated at least twice. Statistical analysis was performed in Prism using unpaired *t* test.

### Quantification of ABA

Material (about 100 mg fresh/dry weight) was suspended in 80% methanol-1% acetic acid containing internal standards and mixed by shaking during 1 h at 4 °C. The extract was kept a −20 °C overnight and then centrifuged, and the supernatant was dried in a vacuum evaporator. The dry residue was dissolved in 1% acetic acid and passed through an Oasis HLB (reverse phase) column as described in Seo et al. (2011). The dried eluate was dissolved in 5% acetonitrile-1% acetic acid, and the hormone was separated using an autosampler and reverse phase UHPLC chromatography (2.6 *µ*m Accucore RP-MS column, 100 mm length × 2.1 mm i.d.; Thermo Fisher Scientific) with a 5% to 50% acetonitrile gradient containing 0.05% acetic acid, at 400 *µ*L/min over 21 min. ABA was analyzed with a Q-Exactive mass spectrometer (Orbitrap detector; Thermo Fisher Scientific) by targeted selected ion monitoring (SIM). The concentration of hormone in the extracts was determined using embedded calibration curves and the Xcalibur 4.0 and TraceFinder 4.1 SP1 programs. The internal standard for quantification of ABA was the deuterium-labeled hormone (^2^H_6_-ABA).

### Statistical analysis

Two-tailed Student's *t* test was performed whenever 2 groups were compared. Statistical significance was determined at *P* < 0.05 unless otherwise indicated. A Kruskal–Wallis test followed by a Mann–Whitney *U* test was performed to assess statistical differences when comparing more than 3 groups, and differences were established with a *P* < 0.05.

### Accession numbers

Sequence data from this article can be found in the GenBank/EMBL data libraries under accession numbers NM_127411 (*HB21*), NM_119838 (*HB40*), and BT024847 (*HB53*).

## Supplementary Material

kiae234_Supplementary_Data

## Supplementary data

The following materials are available in the online version of this article.

**Supplementary Figure S1.** *HB21* expression disappears after inflorescence reactivation.

**Supplementary Figure S2.** *HB21* induction forces flower and inflorescence arrest.

**Supplementary Figure S3.** *HB21* mutants.

**Supplementary Figure S4.** Phenotype of *hb* mutants.

**Supplementary Figure S5.** Number of flowers and fruits in *hb53* and *hb40*.

**Supplementary Figure S6.** *HB21* induction triggers ABA accumulation.

**Supplementary Figure S7.** ABA treatment phenotypes.

**Supplementary Figure S8.** ABA-azide test.

**Supplementary Methods S1.** ABA-azide chemical synthesis.

**Supplementary Table S1.** Final list of DEGs by HB21 induction.

**Supplementary Table S2.** GO analysis of downregulated genes by HB21.

**Supplementary Table S3.** GO analysis of upregulated genes by HB21.

**Supplementary Table S4.** GO analysis of regulated genes by HB21 and AP2.

**Supplementary Table S5.** Primers used in this work.

## Dive Curated Terms

The following phenotypic, genotypic, and functional terms are of significance to the work described in this paper:

HB53 Gramene: AT5G66700
HB53 Araport: AT5G66700

NCED3 Gramene: AT3G14440
NCED3 Araport: AT3G14440

AP2 Gramene: AT4G36920
AP2 Araport: AT4G36920

NCED4 Gramene: AT4G19170
NCED4 Araport: AT4G19170

FUL Gramene: AT5G60910
FUL Araport: AT5G60910

pyl1 Gramene: AT5G46790
pyl1 Araport: AT5G46790

HB21 Gramene: AT2G18550
HB21 Araport: AT2G18550
