# Supplementary material for: Transcription factors HB21/40/53 trigger inflorescence arrest through abscisic acid accumulation at the end of flowering
Source: Plant Physiol. 2024 Apr 26;195(4):2743–56. doi: 10.1093/plphys/kiae234 (PMC11288733; doi:10.1093/plphys/kiae234)
Supplement: kiae234_Supplementary_Data [file kiae234_supplementary_data.zip › PP2023RA01998R1_Supplementary_Data.pdf]

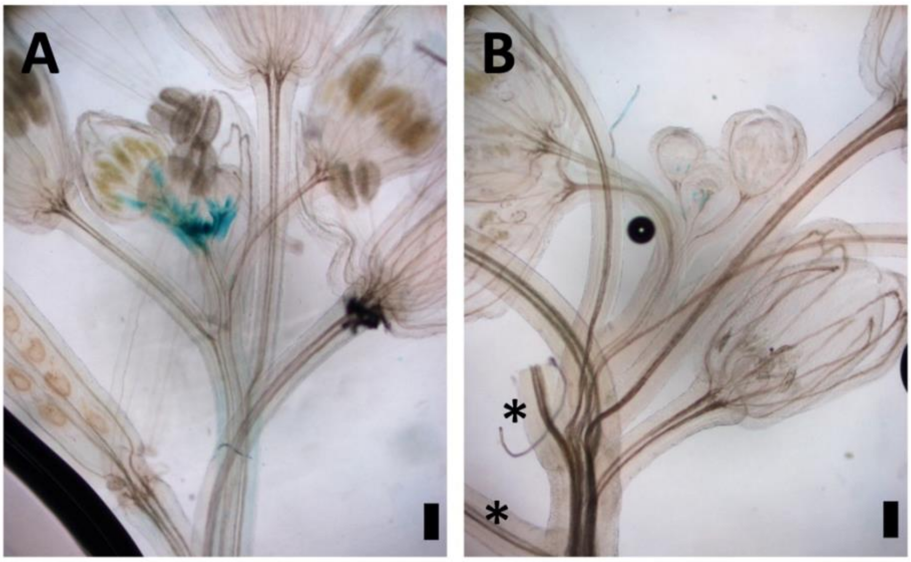

Supplementary Figure S1: ***HB21*** **expression disappears after inflorescence reactivation.** **(A)** *proHB21::GUS* signal is detected in arrested inflorescences. **(B)** *proHB21::GUS* signal disappears after inflorescence reactivation by fruit pruning. Asterisks indicate removed fruits. Black bars represent 200 µm.

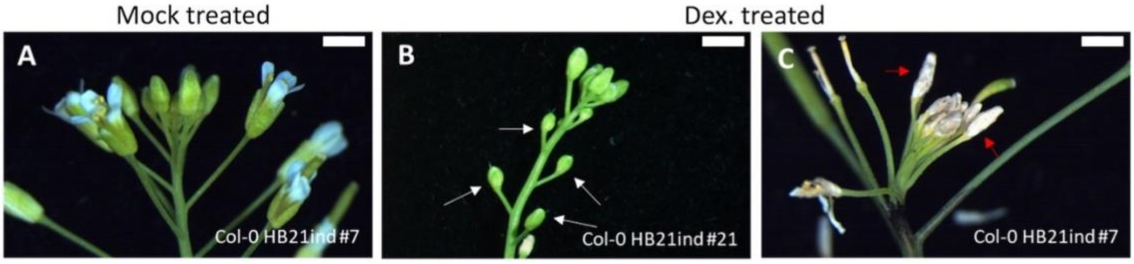

Supplementary Figure S2: *HB21* induction forces flower and inflorescence arrest. Effect of the induction of *HB21* in proliferating inflorescences of WT at 2wab. **(A)** WT *HB21*ind strong #7 line plant, mock-treated, shows normal development **(B)** WT *HB21*ind mild line #21 plant after Dexamethasone (Dex.) treatment, where floral buds are arrested. **(C)** WT *HB21*ind strong #7 line after Dex. treatment, where arrested inflorescence and floral bud senescence are observed. White arrows point to arrested floral buds. Red arrows point to senesced floral buds. Bars represent 1,5mm.

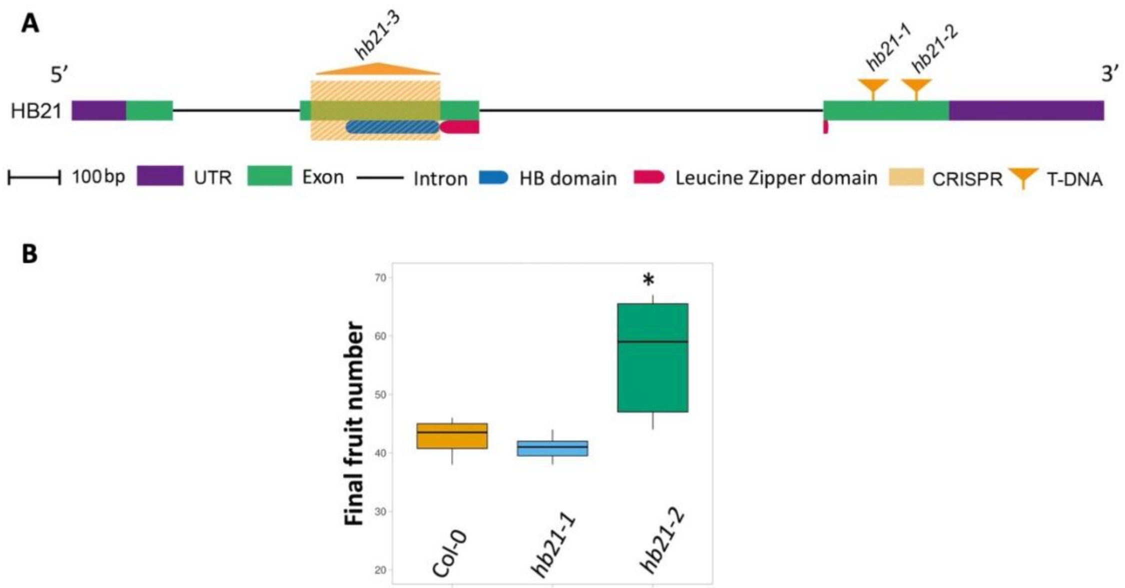

Supplementary Figure S3: ***HB21* mutants. (A)** Schematic representation of *HB21* gene indicating the position of the 3 different alleles used in this work. Purple boxes indicate UTRs, green boxes indicate exons and black line indicates introns. Blue and red boxes indicate the HB and Leucine Zipper domains respectively. Orange box indicates CRISPR deletion and triangles indicates T-DNA insertions and CRISPR deletion. **(B)** Final number of fruits produced by the wild-type Columbia-0 (Col-0) ecotype and the *hb21-1* and *2* mutants. Center lines in the boxplot represent the median and box limits, upper and lower quartiles; whiskers, 1.5x interquartile range. For each group, the sample size was > 10. Black asterisk indicates significant differences (*P < 0.05) respect to the wild type according to the Student's t test.

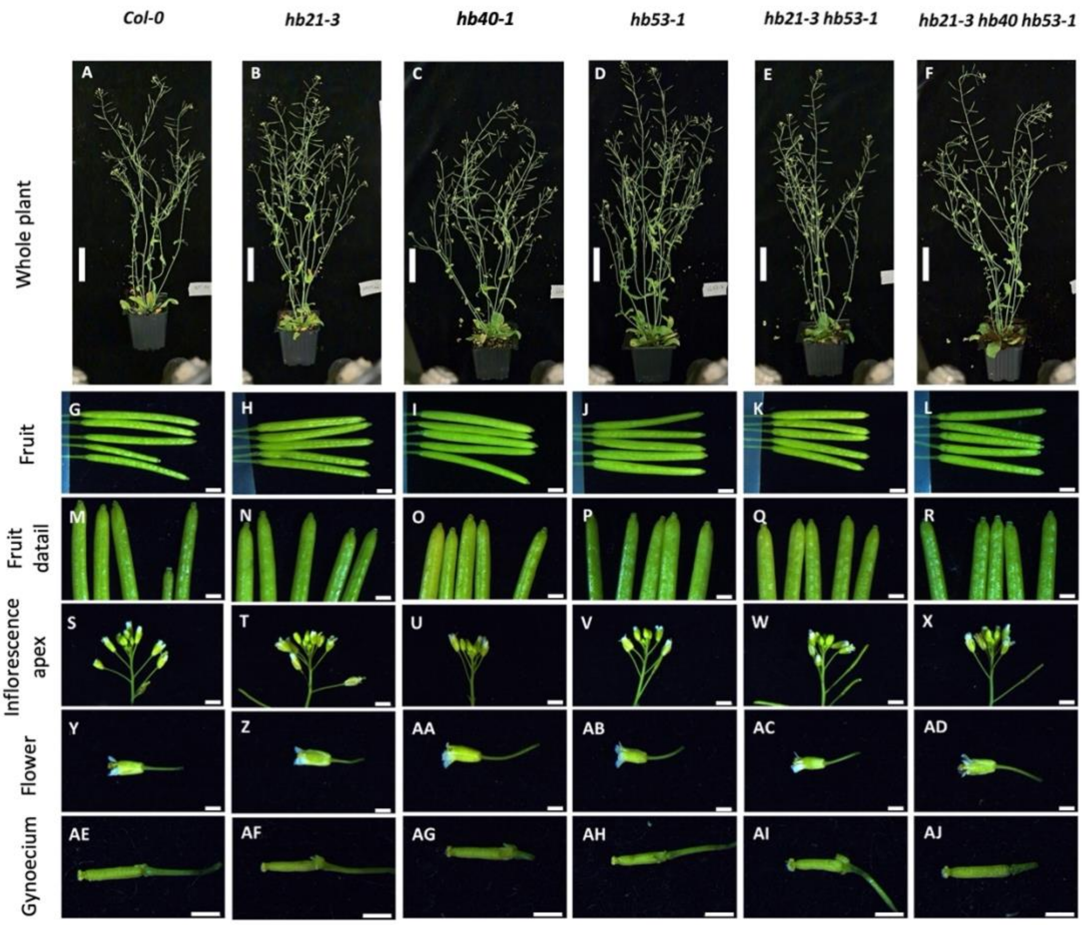

Supplementary Figure S4: Phenotype of *hb* mutants. No differences during development were observed in the *hb* mutants respect to the wild-type plants. **(A-F)** Whole plant phenotype. White bars represent 5 cm. **(G-L)** Fruit phenotype. White bars represent 1 mm**.** **(M-R)** Fruit detail phenotype. White bars represent 0,5 mm. **(S-X)** Inflorescence apex phenotype. White bars represent 1 mm. **(Y-AD)** Flower phenotype. White bars represent 0,5 mm. **(AE-AJ)** Gynoecium phenotype. White bars represent 0,5 mm.

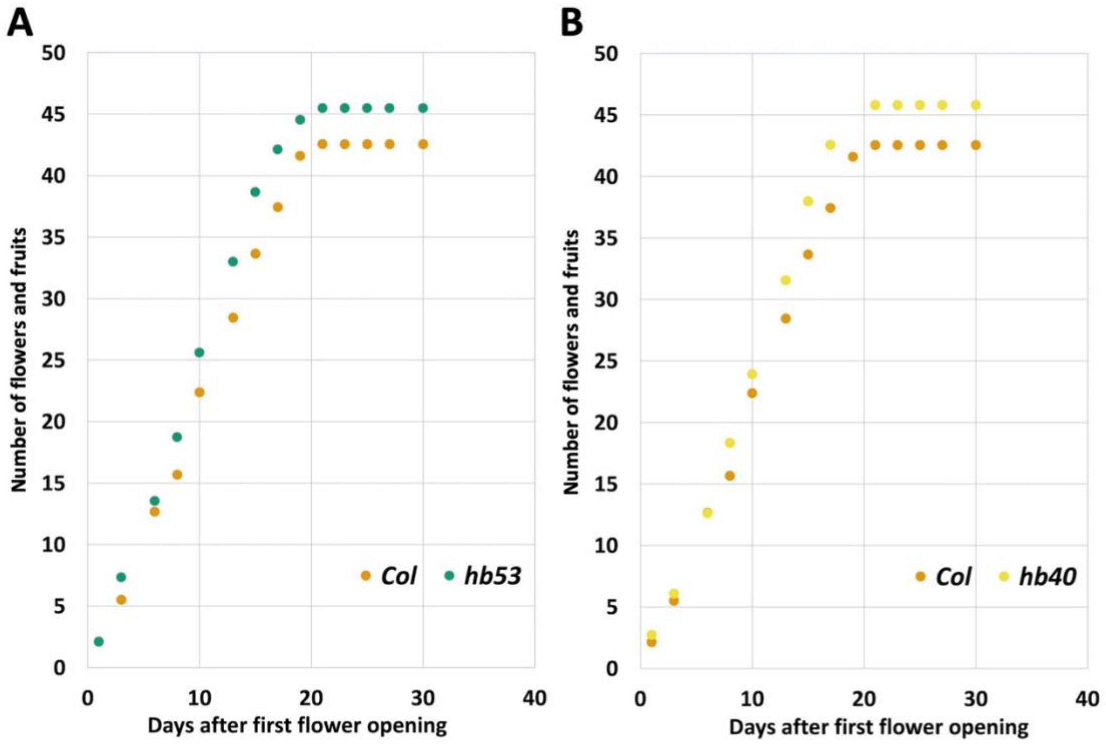

Supplementary Figure S5: Number of flowers and fruits in *hb53* and *hb40.* Number of opened flowers and fruits produced over time by the main inflorescence of WT and *hb53* (A), and *hb40* (B). Dots represent the average of at least 10 plants.

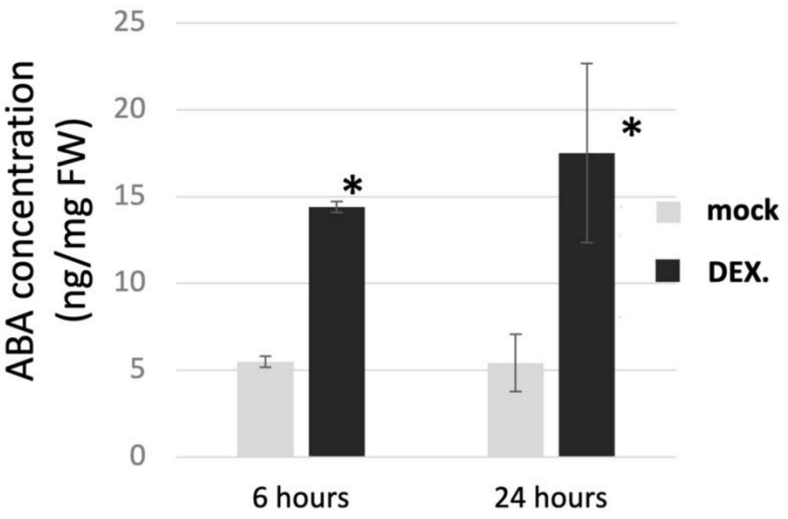

Supplementary Figure S6. ***HB21*** **induction triggers ABA accumulation.** ABA levels increases in the inflorescence apexes of the *HB21* inducible line after 6 and 24 hours of DEX treatment. Black asterisk indicates significant differences (*P < 0.05) respect to the mock treated plants according to the Student's t test . Error bars represent the SD of three biological replicates. FW means fresh weight.

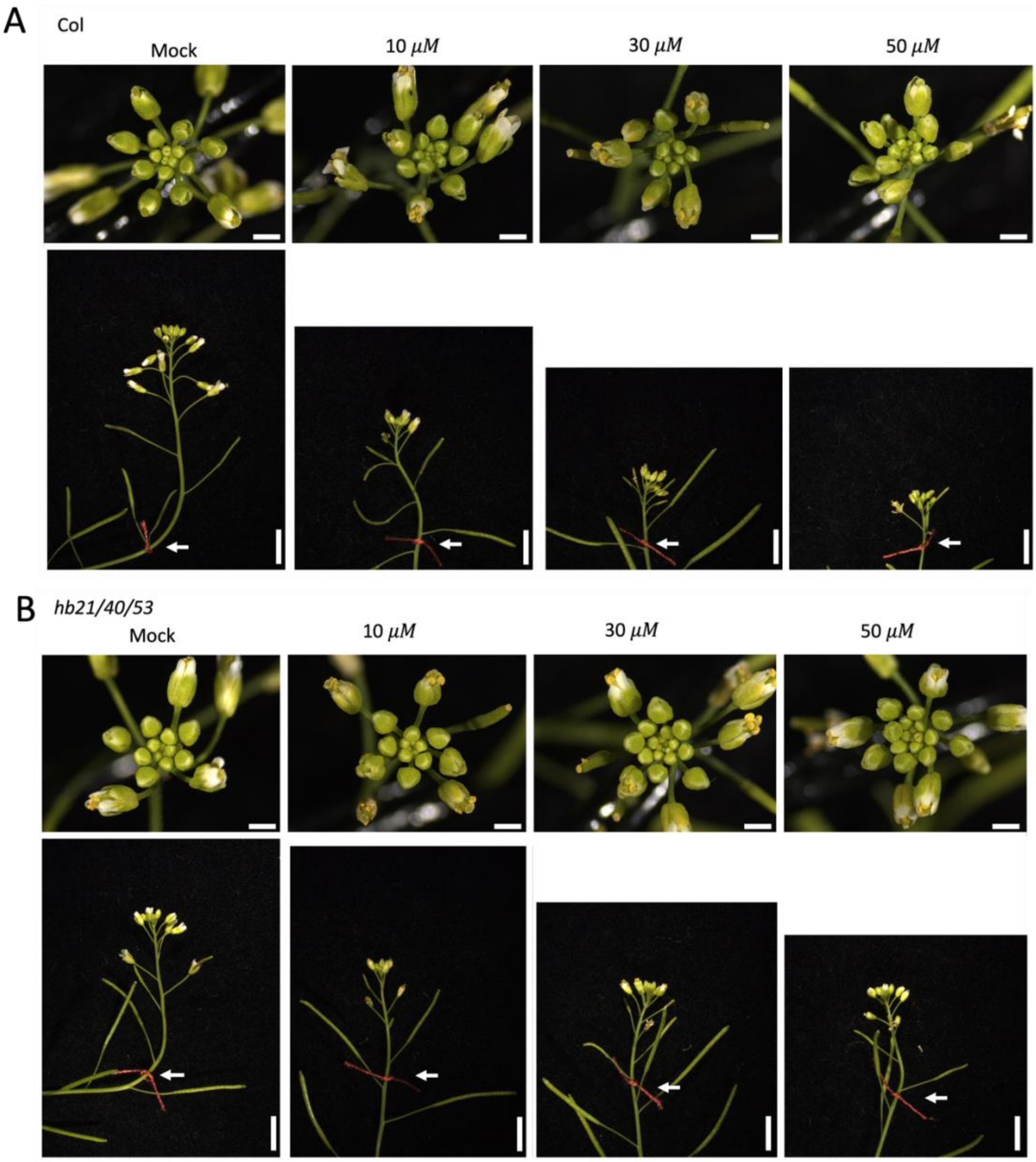

Supplementary Figure S7. ABA treatment phenotypes. (A) Wild-type plant phenotypes 5 days after the treatment. Above panels are inflorescence apexes. Bottom panels are inflorescences. From left to right are represented mock, 10 µM, 30 µM and 50 µM treatments. **(B)** *hb21/40/53* triple mutant plant phenotypes 5 days after the treatment. Above panels are inflorescence apexes. Bottom panels are inflorescences. From left to right are represented mock, 10 µM, 30 µM and 50 µM treatments. White arrows indicate the point were treatments started. White bars in above panels represents 1mm, while white bars in bottom panels represent 5mm.

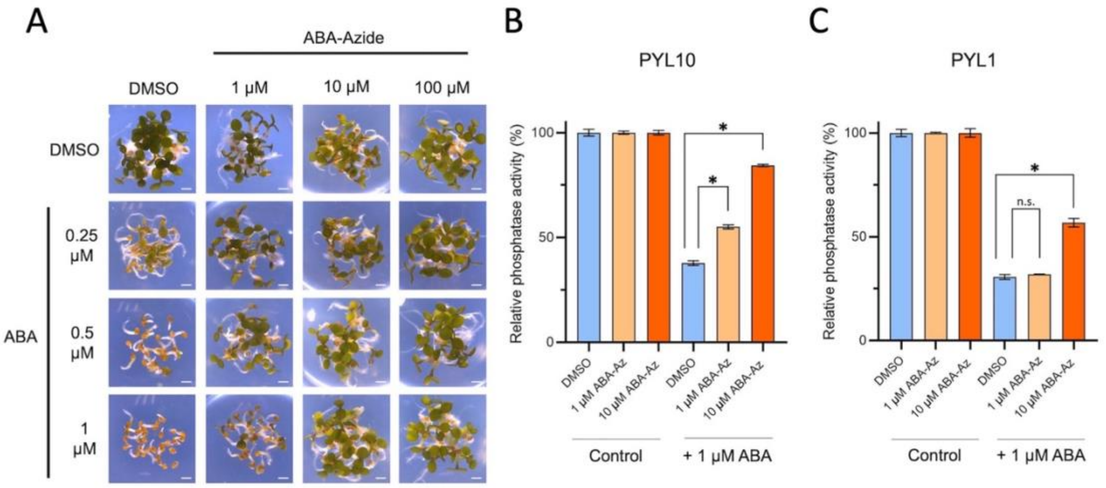

Supplementary Figure S8. ABA-azide test. (A) Seedling establishment of Col-0 seeds treated with ABA and/or ABA-azide at different concentrations. Pictures were taken on day 3. White bars represent 1mm. (B and C) Relative phosphatase activity of recombinant ΔNHAB1 in the presence of 1 µM ABA and 1 µM or 10 µM ABA-azide, and the ABA receptors PYL10 (B) and PYL1 (C). Each value of the treatment group ("+1 µM ABA") is relative to a value of the control group, represented with the same color. Values represent means ± SD of three replicates. * indicates p-value<0.0001 by Student's t-test.

Supplementary Methods 1:
ABA-azide chemical synthesis:

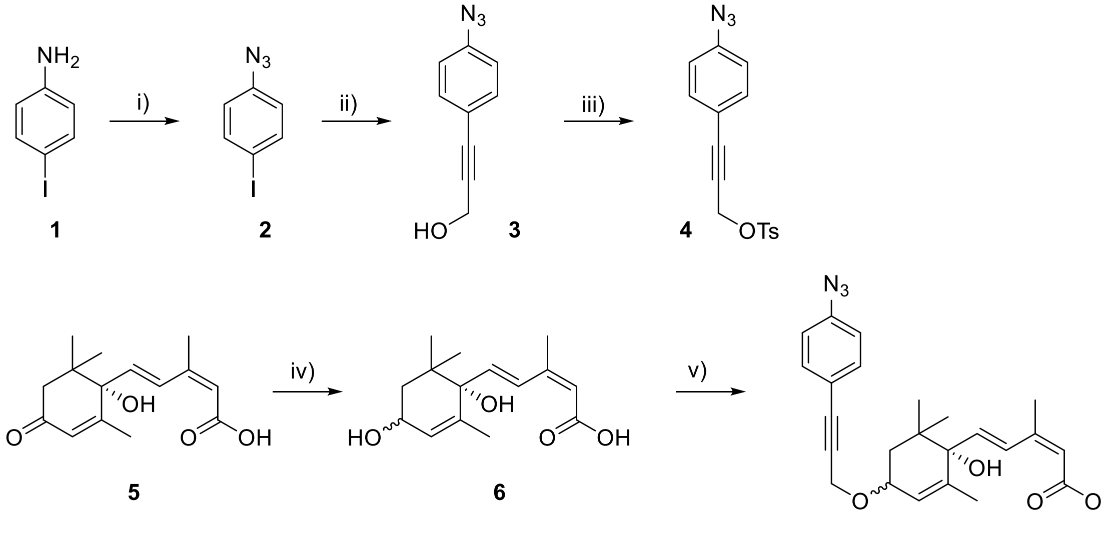

Scheme 1. Synthesis of compound **7**. Reagents and conditions: i) $NaNO_2$, HCl 4M, NaN_3, 0 °C to r.t, 4-12 h, 64 %; ii) Pd $(Ph_3)_2Cl_2$, propargyl alcohol, CuI, Et_3N, r.t, 16 h, 72 %; iii) TsCl, KOH, Et_2O, 0 °C, 2-4 h, 100 %; iv) $CeCl_3{\cdot}6H_2O$, $NaBH_4$, MeOH, 0 °C, 1 h, 54-87 %; v) NaH (60 %), **4**, THF, 0 °C to r.t, 48 h, 22 %.

i) Sodium nitrite (6.3 g, 91 mmol) was added slowly in portions over a period of 15 mins to a solution of 4-iodoaniline (5 g, 22.83 mmol) in HCl 4M (76 mL) in an ice bath. After 30 min of stirring at 0 °C, sodium azide was added in portions and the resulting mixture was stirred for 4-12 h. Then the mixture was poured into H_2O $_{(dest)}$ (50 mL) and was extracted with AcOEt (3x 150 mL). The combined organic phases were washed with H_2O $_{(dest)}$ (40 mL), followed by brine (40 mL), dried over Na_2SO_4, filtered and concentrated under vacuum. The reaction crude was purified by Silica Gel Column Chromatography (Hex/AcOEt gradient from 10:0 to 9:1) to afford the desired product.

ii) In a dried flask it was prepared a mixture of the compound (**2**, 2 g, 8.16 mmol), Pd $(Ph_3)_2Cl_2$ (96 mg, 0.136 mmol), propargyl alcohol (0.39 mL, 6.8 mmol), CuI (52 mg, 0.27 mmol) and freshly distilled Et_3N (27 mL) under nitrogen atmosphere. The resulting mixture was stirred for 16 h at room temperature and then it was quenched with saturated ammonium chloride (20 mL) and extracted with AcOEt (3x 40 mL). The combined organic phases were washed with brine (20 mL), dried over $MgSO_4$, filtered and concentrated under vacuum. The reaction crude was purified by Silica Gel Column Chromatography (Hex/AcOEt gradient from 10:0 to 6:4) to afford the desired product.

iii) To a solution of compound (**3**, 428 mg, 2.47 mmol) in dry ether (3.9 mL) at 0 °C under nitrogen atmosphere, 4-toluenesulfonyl chloride (565 mg, 296 mmol) was added. After a few minutes, crushed potassium hydroxide (776 mg, 13.83 mmol) was added to the solution and stirred at the same temperature until the complete conversion of the starting material. Then the reaction was quenched with H_2O $_{(dest)}$ (5 mL) and extracted with Et_2O (3x 10 mL). The combined organic layers were dried over $MgSO_4$, filtered and evaporated under low vacuum. The reaction crude was used in the next step without further purification.

iv) To a solution of abscisic acid (183 mg, 0.69 mmol) in dry methanol (8.3 mL) in an ice bath under nitrogen atmosphere, cerium (III) chloride heptahydrate (722 mg, 1.94 mmol) was added. After stirring the mixture for 10 min at the same temperature, sodium borohydride (204 mg, 5.4 mmol) was added in small portions. The reaction was stirred for 62 min at 0 °C and then was quenched with saturated ammonium chloride solution (4 mL). The solvent was evaporated and the aqueous phase was acidified until pH ≈ 2 by the addition of HCl 1M. The resulting mixture was extracted with AcOEt (3x 9 mL). The combined organic layers were washed $H_2O_{(dest)}$ (8 mL) and brine (8 mL), dried over $MgSO_4$, filtered and concentrated under vacuum. The reaction crude was purified by Silica Gel Column Chromatography (Hex/AcOEt/AcOH gradient from 10:0:0.1 to 6:4:0.1) to afford the desired product.

v) With stirring at 0 °C, sodium hydride (60 % in oil, 44 mg, 1.1 mmol) was added to a solution of compound **6** in distilled THF (3 mL) under nitrogen atmosphere. After being stirred for 30 min at room temperature, a solution of **4** in THF (2 mL) was added to the first suspension. The mixture was stirred for 48 h at room temperature and then was quenched with HCl 1M (1.2 mL), it was diluted with $H_2O_{(dest)}$ (15 mL) and extracted with AcOEt (3x 15 mL). The combined organic layers were washed with brine, dried over $MgSO_4$, filtered and concentrated under vacuum. The reaction crude was purified by Silica Gel Column Chromatography (Hex/AcOEt/AcOH gradient from 10:0:0.1 to 1:1:0.1) to afford the desired product.

2: brown solid, 64 % yield. ^{1}H NMR (300 MHz, $CDCl_3$) δ/ppm = 7.64 (d, *J* = 8.8 Hz, 2H), 6.78 (d, *J* = 8.8 Hz, 2H).^{13}C NMR (75 MHz, $CDCl_3$) δ/ppm = 140.11, 138.83, 121.16, 88.34.
3: brown solid, 72 % yield. ^{1}H NMR (400 MHz, $CDCl_3$) δ/ppm = 7.41 (d, *J* = 8.7 Hz, 2H), 6.96 (d, *J* = 8.7 Hz, 2H), 4.49 (s, 2H). ^{13}C NMR (101 MHz, $CDCl_3$) δ/ppm = 140.32, 133.23, 119.13, 87.56, 85.03, 77.16, 51.64.
4: brown solid, 100 % yield. ^{1}H NMR (400 MHz, $CDCl_3$) δ/ppm = 7.84 (d, *J* = 8.3 Hz, 2H), 7.32 (d, *J* = 8.5 Hz, 2H), 7.28 – 7.21 (m, 2H), 6.93 (d, *J* = 8.7 Hz, 2H), 4.93 (s, 2H), 2.40 (s, 3H). ^{13}C NMR (101 MHz, $CDCl_3$) δ/ppm = 145.17, 141.03, 133.45, 129.93, 128.30, 119.08, 118.02, 88.34, 81.05, 77.16, 58.65, 21.72. HRMS (ESI) calcd. for $C_{16}H_{13}N_3O_3NaS^+$ $[M+Na]^+$ = 350.0575, found $[M+Na]^+$ = 350.0576 (0.3 ppm).
6: colorless solid, 54-87 % yield. ^{1}H NMR (300 MHz, $CDCl_3$) δ/ppm = 7.61 (d, *J* = 16.4 Hz, 1H), 6.08 (d, *J* = 16.4 Hz, 1H), 5.72 (s, 1H), 5.69 (s, 1H), 4.24 (tdd, *J* = 6.8, 4.5, 2.2 Hz, 1H), 2.04 (d, *J* = 1.2 Hz, 3H), 1.81 (dd, *J* = 13.5, 6.9 Hz, 1H), 1.68 – 1.65 (m, 3H), 1.64 – 1.57 (m, 1H), 1.04 (s, 3H), 0.91 (s, 3H). ^{13}C NMR (101 MHz, $CDCl_3$) δ/ppm = 168.3, 150.6, 140.1, 138.2, 127.4, 126.6, 117.0, 78.9, 65.0, 43.5, 39.6, 24.2, 21.8, 20.0, 16.8. HRMS (ESI) calcd. for $C_{15}H_{21}O_4^-$ $[M-H]^-$ = 265.1440, found $[M-H]^-$ = 265.1442 (0.8 ppm).
7: brown solid, 22 % yield. ^{1}H NMR (300 MHz, $CDCl_3$) δ/ppm = 7.74 (d, *J* = 16.0 Hz, 1H), 7.42 (d, *J* = 8.7 Hz, 2H), 6.97 (d, *J* = 8.7 Hz, 2H), 6.21 (d, *J* = 16.0 Hz, 1H), 5.72 (d, *J* = 6.4, 1H), 5.69 (d, *J* = 5.3 Hz, 1H), 4.43 (d, *J* = 2.9 Hz, 2H), 4.24 (ddd, *J* = 8.9, 4.4, 2.2 Hz, 1H), 2.02 (d, *J* = 1.1 Hz, 3H), 1.92 – 1.85 (m, 1H), 1.77 – 1.70 (m, 1H), 1.70 – 1.64 (m, 3H), 1.06 (s, 3H), 0.92 (s, 3H). ^{13}C NMR (75 MHz, $CDCl_3$) δ/ppm = 169.48, 152.46, 140.47, 140.46, 139.36, 133.41, 126.66, 124.72, 119.37, 119.17, 116.64, 86.01, 85.39, 79.37, 77.36, 77.16, 72.56, 56.29, 40.83, 39.82, 25.44, 22.83, 21.70, 17.84. HRMS (ESI) calcd. for $C_{24}H_{27}N_3NaO_4^+$ $[M+Na]^+$ = 444.1899, found $[M+Na]^+$ = 444.1900 (0.2 ppm).

***HB21* CrispR** (Wang et al., 2015)		
name	sequence	purpose
oVSG077	ATATATGGTCTCGATTGAGAAGCAAAACCAACACGGGTT	RNAg cloning in pHEE401: DT1-BsF_HB21
oVSG078	TGAGAAGCAAAACCAACACGGGTTTTAGAGCTAGAAATAGC	RNAg cloning in pHEE401: DT1-F0_HB21
oVSG079	AACCGACTCGTTTGTTCTTCCACAATCTCTTAGTCGACTCTAC	RNAg cloning in pHEE401: DT2-R0_HB21
oVSG080	ATTATTGGTCTCGAAACCGACTCGTTTGTTCTTCCACAA	RNAg cloning in pHEE401: DT2-BsR_HB21

**RT-qPCR primers**		
name	sequence	purpose
qHB21 5'	TCGCCGTCTGGTTCCAA	qRT-PCR of *HB21*
qHB21 3'	CATCCTCGACTCGTTTGTTCTTC	qRT-PCR of *HB21*
qHB40 5'	GGAGGAGGAAGAAGACCAAAGG	qRT-PCR of *HB40*
qHB40 3'	ACAAACCGTTGCCTCCATCT	qRT-PCR of *HB40*
qHB53 5'	GGAGTCAGGGAGGAAGGAGAA	qRT-PCR of *HB53*
qHB53 3'	CCACCTGTCTCGGGTCAAGA	qRT-PCR of *HB53*
TIP41 F	GTGAAAACTGTTGGAGAGAAGCAA	qRT-PCR reference
TIP 41 R	TCAACTGGATACCCTTTCGCA	qRT-PCR reference

Supplementary Table S5: Primers used in this work.

References:

Wang Z-P, Xing H-L, Dong L, Zhang H-Y, Han C-Y, Wang X-C, Chen Q-J (2015) Egg cell-specific promoter-controlled CRISPR/Cas9 efficiently generates homozygous mutants for multiple target genes in Arabidopsis in a single generation. Genome Biol **16**: 144
