# Supplementary material for: Transcription factors HB21/40/53 trigger inflorescence arrest through abscisic acid accumulation at the end of flowering
Source: Plant Physiol. 2024 Apr 26;195(4):2743–56. doi: 10.1093/plphys/kiae234 (PMC11288733; doi:10.1093/plphys/kiae234)
Supplement: kiae234_Supplementary_Data [file pp.01998.2023-s07.pdf › pp.01998.2023-s07.pdf]

Plant Physiology®

HB21/40/53 promote inflorescence arrest through ABA accumulation at the end of flowering

Verónica Sánchez-Gerschon, Irene Martínez-Fernández, Maria González-Bermúdez, Sergio de la Hoz-Rodríguez, Florenci González, Jorge Lozano-Juste, Cristina Ferrandiz, and Vicente Balanzà

Review Timeline:

Submission Date:	14-Dec-2023
Editorial Decision:	24-Jan-2024
Revision Received:	28-Feb-2024
Accepted:	01-Apr-2024

24-Jan-2024

Dr. Vicente Balanzà
Instituto de Biologia Molecular y Celular de Plantas
Development
Valencia 46022
Spain

RE: HB21/40/53 promote inflorescence arrest through ABA accumulation at the end of flowering

Dear Dr. Balanzà:

We have received reviews of your manuscript, which you may view at the end of this letter. On the basis of the advice received and my own evaluation, I believe that, pending revision, this article may ultimately be acceptable for publication in Plant Physiology.

I'm pleased to inform you that both reviewers highly evaluated this work. However, they suggested some points for further improvements. In particular, both reviewers pointed out that Fig. 3 needs to be improved by adding quantitative data and a more precise description. Please carefully study their comments and revise the manuscript.

When you are ready to submit your revision, click the below link and find the red arrow under your Author Tasks heading for this revision submission.

If you want to visit this version of the manuscript, you may click the link below and then click on the folder that reads "Post Decision Manuscripts".

Link Not Available

When uploading the revised version of this article, please be sure to include as complete an answer as possible to the referees' criticisms during the first round of reviews in the "response to reviewers" section.

Plant Physiology considers a revision for up to 60 days after a manuscript has been provisionally accepted; limited extension of this timeframe is at the discretion of the Monitoring Editor. A reminder notice will be sent to you 2 weeks before the deadline, at which time you may choose to contact the editorial office to request an extension; if so, please provide a justification for your request. After the expiration of this deadline, the article would need to be submitted as a resubmission and would be handled as such.

Thank you very much for giving us an opportunity to review this work. I look forward to receiving the next version.

Sincerely,

Plant Phys Board of Editors

Want to add this revision deadline to your calendar? Click below!

-- Reviewer comments:
Reviewer #1 (Comments for the Author):

This manuscript describes the role of the HB21/HB40/HB53 transcription factors in the regulation of end-of-flowering in Arabidopsis. The results presented here follow on logically from earlier studies by the same authors, and provide clear evidence these genes regulate the specific developmental process of 'floral arrest' by controlling abscisic acid metabolism. I found the

data convincing and clear, and overall, I think this is an excellent manuscript.

However, I do there think are a few major issues which the authors should address in order to make this manuscript easier-to-read, easier-to-understand, and more convincing.
-- Tom Bennett

Major Issues:
1) Terminology.
The main finding of the authors is that HB21 etc. control the "developmental block of the unpollinated flower buds" during end of flowering. The authors use this unnecessarily wordy description throughout the text. However, this process has already been described in Walker et al 2023 (as the authors acknowledge), where it was described as "floral arrest". It would be better if the authors just used the term already present in the literature for this process. This would make the manuscript more consistent with previous work, would help the reader understand that the two manuscripts describe the same phenomenon, and would also just make it much easier to read the text.

2) Confusion/conflation between inflorescence meristem arrest and floral arrest (and also proliferative arrest).
The authors are very clear in their conclusion that HB21/HB40/HB53 only regulate floral arrest and not inflorescence meristem activity or arrest, and that is absolutely what their data show. However, throughout the text, there is considerable confusion or conflation between these processes. The authors also frequently use the term "proliferative arrest" to mean "meristem arrest", which again just introduces confusion for the reader -- it would be better to stick to specific terms. The following examples illustrate what I mean, and the text should be changed to avoid creating confusion for the reader.
a) Line 121 - "The inflorescence arrest at the end of the flowering phase has been interpreted as a dormancy state." -- this should say "the inflorescence meristem arrest"
b) Line 199 - "The inflorescence proliferative arrest" --> This should just say "inflorescence meristem arrest"
c) Line 246 - "HB21 protein could be triggering the dormant state associated to proliferative arrest at the end of the flowering period." -- but the authors have shown HB21 isn't expressed in the IM, so this isn't what the data suggest. And further more, the authors go on to clearly demonstrate that HB21 doesn't regulate IM arrest. So this statement is unnecessarily confusing.
d) Line 326 - "reaching the highest expression level in arrested meristems" - this should say "arrested apices". The authors did not look specifically at meristems, and they show the HB genes aren't expressed in the meristem anyway.
e) Line 336 - "that the three HB genes might also act redundantly in the control of proliferative arrest at the end of the flowering phase." Again, proliferative arrest is unnecessarily vague here. And again, assuming that proliferative arrest = meristem arrest, the data don't suggest this, because the HB genes aren't expressed in the inflorescence meristem.
f) Line 442 - "AP2 and HB21 exert opposite effects on meristem arrest" - No they don't, HB21 doesn't exert any effect on meristem arrest.
g) Line 639 - " The end of flowering is characterized by two main events: developmental block of floral buds and apex arrest". Here, apex arrest should say meristem arrest.

3) Figures & Figure Legends
The graphs in Figures 4, 5 and 7 could be improved. The colours used for the different mutants are not a pleasant combination - it would actually be preferable for these graphs to be grayscale or have fewer colours. The numbers on the y axis for these graphs are very small and difficult to read Figure 4A in particular is very difficult due to the combination of colours and symbols used.
Figure legends for these figures, and for supplementary figure 4 and 6 have no sample sizes in them. These need to be added.

4) Inducible HB21 data (Figure 3)
The data presented in Figure 3, regarding the inducible HB21 lines consists of 3 pictures, with no quantification of any trait, and no description of how often these effects were seen. Just once in 1 plant? With 100% effectiveness is many plants? In x% of treated plants? As currently presented, these data are not sufficient to draw any conclusion.

Given the subsequent analysis of hb21 mutants, I am prepared to accept that "In line #21 plants (mild induction), Dex treatment induced the development arrest of unpollinated floral buds". However, the conclusion that "In the other hand, line #7 (strong induction) showed a clear response to treatment on the whole meristem apex, inducing floral bud senescence and inflorescence arrest" is clearly not sustainable based on the date presented. What the data in Figure 3C show is not a naturally arrested inflorescence, but an inflorescence apex that has been killed by misexpression of HB21. This is not good evidence for the role of HB21.

I think that these data either need i) removing from the paper (they are not really needed, given the analysis of the mutants), ii) relegating to the supplementary data or iii) proper analysis and quantification, if the authors want to retain it as a primary figure. But either way, the conclusion regarding line #7 needs modifying in the text.

5) Manuscript length
At 700 lines of text, the manuscript seems rather long for the amount of primary data being presented. The section from lines 413-466 , describing the results of the transcriptomic analysis, could be dramatically condensed, since the justification of ABA as a target for further investigation does not need 50 lines of text, given the well-described role of HB21/40/53 in regulating ABA.

Minor issues:
1) Line 61: "The flowering period is the time during which a plant produces flowers" - this is rather a statement of the obvious.
2) Line 66: "The inflorescence meristem, then, actively generates flowers" --> The inflorescence meristem then actively generates flowers
3) Line 125/126: IM arrest is reversible in Ler, but not in Col-0 (Walker et al, 2023), so this statement needs to be qualified - it is not universally true.
4) Line 138: " While the role of ABA in axillary bud dormancy is well established," -- I'm not sure that the role of ABA in axillary bud dormancy is really that well established (it is a very mild effect at best), but either way, this statement need supporting with references.
5) Line 144, Line 253: "associated to" --> associated with
6) Line 108, Line 176, Line 179, Line 181, Line 200, Line 211: In all of these places, the inflorescence meristem is instead referred to as a shoot apical meristem (SAM). This is technically correct, but confusing, because "inflorescence meristem" is used elsewhere for the same structure, and by convention, SAM usually refers to vegetative meristems. In would be better to use IM throughout.
7) Line 200: "where is no longer" --> "where it is no longer"
8) Line 249: dexamethasone shouldn't have a capital letter, it isn't a proper noun.
9) Line 299-301: ". This analysis indicated that the absence of HB21 could affect the number of flowers produced before arrest increasing the flower
production around 10%" -- the data don't really show this, because there is no statistical difference, and the range of the data is completely overlapping. I don't think this is a safe conclusion.
10) Line 305: "preliminary" --> previous
11) Line 372: "doble" --> double
12) Line 408, Line 559 : "Mann Whitney U's test" --> Mann Whitney U test
13) Line 411: "arrest controlling" --> arrest by controlling
14) Line 482-484: I think this is primary data and should be in a main figure, not in a supplemental.
15) Line 508: "significative" --> significant
16) Line 524 and line 526: "respect to" --> with respect to
17) "This outgrowth block mediated by BRC1 is highly dependent on ABA (van Es et al., 2023)". This is not what the data in van Es show -- indeed, they show that the effect of BRC1 is not highly dependent on ABA.
18) Line 612: "afecting mainly to flower development" --> mainly affecting flower development
19) Line 614-615: there is evidence that CK specifically counteracts the repressive effect of ABA in bud outgrowth. In fact, CK represses BRC1, and therefore indirectly represses ABA and many other BRC1-regulated processes. This sentence needs changing.
20) Line 639-640: The data in Walker et al, 2023 show that floral arrest and inflorescence meristem arrest are indeed processes that occur close together in time (Figure 1A). It takes a long time for the effects of floral arrest to become visible, but the process is clearly initiated much earlier. However, the IM arrest and floral arrest processes do not seem to be completely synchronous.
21) Line 667-675: This discussion of the possible relationship between arrest processes in cereals and Arabidopsis is certainly very interesting. However, it also represents a massive over-simplification. The arrest processes are very different between different cereals, and in wheat for instance, there is no pre-anthesis tip degeneration (instead, the spike meristem is converted to a spikelet meristem). This discussion needs far more space that it can be given here, since it also needs actually descriptions of the shoot architecture for different cereals in order for the reader to understand it). So I would suggest dramatically shortening this paragraph instead, and just noting that Grassy Tillers has been implicated in the end of flowering in barley, without going into any of the morphological detail.

Reviewer #2 (Comments for the Author):

In this manuscript, Sánchez-Gerschon et al. aimed to understand the molecular mechanism of inflorescence arrest at the end of flowering in Arabidopsis thaliana. The authors focused on HOMEOBOX PROTEIN 21/40/53 (HBs) as key regulators in the control of inflorescence arrest and showed that the three HBs play an important role in the control of inflorescence development through ABA biosynthesis and response. This manuscript will provide important information on how the inflorescence meristem arrests at the end of flowering.

Major comments:
1. The expression data shown in Figures 1 and 2 strongly suggest that HB21 is involved in inflorescence arrest. However, genetic analyses are not sufficient to support the authors' claim. To further clarify the function of HBs in inflorescence arrest, the authors should analyse the expression of WUS-GFP in HB21 ind plants (#21 and #7). Furthermore, the inflorescence phenotype of HB21 ind plants (#21 and #7) is not well characterized (Fig. 3 B/C). Alterations in inflorescence architecture should be more clearly described and discussed using quantitative data.
2. With regard to the HB 40 and HB53 genes, the spatial expression patterns of these genes in the inflorescence should be analysed using a GUS reporter as well as HB21 (González-Grandío et al, 2017). In the discussion part, the authors propose the possibility that the transcripts of the HB genes directly affect the activity of the meristem in a non-cell autonomous manner. The expression data for HB 40/53 in the inflorescence may be helpful in supporting the authors' claims.

3. The three HB genes mentioned in this paper are up-regulated in axillary meristems by the TCP transcription factor BRC1. Are BRC1 or other TCPs involved in the transcriptional activation of HBs in the inflorescence shoot and in inflorescence arrest at the end of flowering? Since we don't know how environmental signals induce the HB genes, if known, it would be good to state.

Minor comments:
1. There are many typographical errors (L170, 186, 189, 229, 315, 366, 368, 487, 488, 489, 490, 538, 633, 696 etc.) in this manuscript. Please correct them in the revised version.

RE: HB21/40/53 promote inflorescence arrest through ABA accumulation at the end of flowering

Dear Dr. Balanzà:

We have received reviews of your manuscript, which you may view at the end of this letter. On the basis of the advice received and my own evaluation, I believe that, pending revision, this article may ultimately be acceptable for publication in Plant Physiology.

I'm pleased to inform you that both reviewers highly evaluated this work. However, they suggested some points for further improvements. In particular, both reviewers pointed out that Fig. 3 needs to be improved by adding quantitative data and a more precise description. Please carefully study their comments and revise the manuscript.

When you are ready to submit your revision, click the below link and find the red arrow under your Author Tasks heading for this revision submission.

If you want to visit this version of the manuscript, you may click the link below and then click on the folder that reads "Post Decision Manuscripts".

https://pphys.msubmit.net/cgi-bin/main.plex?el=A2It3Nsz6A2Fwc6F2A9ftdH3cnCcUxY4LnEAgShJuqdQZ

When uploading the revised version of this article, please be sure to include as complete an answer as possible to the referees' criticisms during the first round of reviews in the "response to reviewers" section.

Plant Physiology considers a revision for up to 60 days after a manuscript has been provisionally accepted; limited extension of this timeframe is at the discretion of the Monitoring Editor. A reminder notice will be sent to you 2 weeks before the deadline, at which time you may choose to contact the editorial office to request an extension; if so, please provide a justification for your request. After the expiration of this deadline, the article would need to be submitted as a resubmission and would be handled as such.

Thank you very much for giving us an opportunity to review this work. I look forward to receiving the next version.

Sincerely,

Plant Phys Board of Editors

Plant Physiology

Dear Editor

We are very pleased that our manuscript could be published in Plant Physiology.
We have studied the reviewers' suggestions and questions and tried to answer them all.
Regarding the Figure 3, where both reviewers had some concerns, we have written again the section, trying to explain better our purpose, and changed the figure. Our aim was to get some evidence of the role of HB21 during inflorescence development and for this we mis-expressed the gene locally in the inflorescence apex. Now, we include the % of T1 plants affected when HB21 is induced and show the inflorescence phenotypes that we observed in these T1 plants. The former figure, representing T3 plants, has been maintained in supplemental material to show that phenotypes are stable. Despite one of the reviewers suggests removing these data, we prefer to keep them as it clearly reflects the impact of HB21 on inflorescence and flower development when expressed ectopically. We believe that the precise role of the HB genes (in place and time) is more clearly defined by the analysis of the simple, double and triple mutant combinations, so figure 3 is more of a complement and, as such, we think that including the T1 data is preferable to repeating the experiment in the original Figure 3. This would take at least two/three months, but the results of this analysis would not modify/affect the message of the manuscript, because the observed phenotypes clearly correlate with the ones observed in the mutants, which are perfectly described in the work.

Respect to the rest of comments we have answered and included in the text most of them when possible.

If you consider that additional changes could be necessary, we will be pleased to introduce them.

Sincerely,

Vicente Balanzà

-- Reviewer comments:
Reviewer #1 (Comments for the Author):

This manuscript describes the role of the HB21/HB40/HB53 transcription factors in the regulation of end-of-flowering in Arabidopsis. The results presented here follow on logically from earlier studies by the same authors, and provide clear evidence these genes regulate the specific developmental process of 'floral arrest' by controlling abscisic acid metabolism. I found the data convincing and clear, and overall, I think this is an excellent manuscript.

However, I do there think are a few major issues which the authors should address in order to make this manuscript easier-to-read, easier-to-understand, and more convincing.
-- Tom Bennett

We really appreciate all the comments and suggestions of Dr Bennett and as well as we consider that them are going to improve our piece of work.

Major Issues:
1) Terminology.
The main finding of the authors is that HB21 etc. control the "developmental block of the unpollinated flower buds" during end of flowering. The authors use this unnecessarily wordy description throughout the text. However, this process has already been described in Walker et al 2023 (as the authors acknowledge), where it was described as "floral arrest". It would be better if the authors just used the term already present in the literature for this process. This would make the manuscript more consistent with previous work, would help the reader understand that the two manuscripts describe the same phenomenon, and would also just make it much easier to read the text.
We have updated the text according to Dr. Bennett suggestion.

2) Confusion/conflation between inflorescence meristem arrest and floral arrest (and also proliferative arrest).
The authors are very clear in their conclusion that HB21/HB40/HB53 only regulate floral arrest and not inflorescence meristem activity or arrest, and that is absolutely what their data show. However, throughout the text, there is considerable confusion or conflation between these processes. The authors also frequently use the term "proliferative arrest" to mean "meristem arrest", which again just introduces confusion for the reader -- it would be better to stick to specific terms. The following examples illustrate what I mean, and the text should be changed to avoid creating confusion for the reader.

a) Line 121 - "The inflorescence arrest at the end of the flowering phase has been interpreted as a dormancy state." -- this should say "the inflorescence meristem arrest"
b) Line 199 - "The inflorescence proliferative arrest" --> This should just say "inflorescence meristem arrest"
c) Line 246 - "HB21 protein could be triggering the dormant state associated to proliferative arrest at the end of the flowering period." -- but the authors have shown HB21 isn't expressed in the IM, so this isn't what the data suggest. And further more, the authors go on to clearly demonstrate that HB21 doesn't regulate IM arrest. So this statement is unnecessarily confusing.
d) Line 326 - "reaching the highest expression level in arrested meristems" - this should say "arrested apices". The authors did not look specifically at meristems, and they show the HB genes aren't expressed in the meristem anyway.
e) Line 336 - "that the three HB genes might also act redundantly in the control of proliferative arrest at the end of the flowering phase." Again, proliferative arrest is unnecessarily vague here. And again, assuming that proliferative arrest = meristem arrest, the data don't suggest this, because the HB genes aren't expressed in the inflorescence meristem.
f) Line 442 - "AP2 and HB21 exert opposite effects on meristem arrest" - No they don't, HB21 doesn't exert any effect on meristem arrest.
g) Line 639 - " The end of flowering is characterized by two main events: developmental block of floral buds and apex arrest". Here, apex arrest should say meristem arrest.

We agree Dr. Bennett comments and have addressed the text to avoid confusion in terminology.

3) Figures & Figure Legends
The graphs in Figures 4, 5 and 7 could be improved. The colours used for the different mutants are not a pleasant combination - it would actually be preferable for these graphs to be grayscale or have fewer colours. The numbers on the y axis for these graphs are very small and difficult to read Figure 4A in particular is very difficult due to the combination of colours and symbols used.
Figure legends for these figures, and for supplementary figure 4 and 6 have no sample sizes in them. These need to be added.

Figures have been modified, increasing the font size of the y axis numbers. We have also indicated in the legend of the figures the sample size of each panel.
In figure 4A we have changed the symbols used and removed the single mutants values of *hb40* and *53* (which are very similar to the hb21 single mutant and now are in a new supplemental figure), in order to make figure 4A more readable. Respect to the colours used, we do not see a problem with the combination of colors, this is a very subjective matter and we prefer to keep it. .

4) Inducible HB21 data (Figure 3)
The data presented in Figure 3, regarding the inducible HB21 lines consists of 3 pictures, with no quantification of any trait, and no description of how often these

effects were seen. Just once in 1 plant? With 100% effectiveness is many plants? In x% of treated plants? As currently presented, these data are not sufficient to draw any conclusion.

Given the subsequent analysis of hb21 mutants, I am prepared to accept that "In line #21 plants (mild induction), Dex treatment induced the development arrest of unpollinated floral buds". However, the conclusion that "In the other hand, line #7 (strong induction) showed a clear response to treatment on the whole meristem apex, inducing floral bud senescence and inflorescence arrest" is clearly not sustainable based on the date presented. What the data in Figure 3C show is not a naturally arrested inflorescence, but an inflorescence apex that has been killed by misexpression of HB21. This is not good evidence for the role of HB21.

I think that these data either need i) removing from the paper (they are not really needed, given the analysis of the mutants), ii) relegating to the supplementary data or iii) proper analysis and quantification, if the authors want to retain it as a primary figure. But either way, the conclusion regarding line #7 needs modifying in the text.

We have tried to solve the concerns of the reviewer. Our intention was to provide qualitative data, more than quantitative, that HB21 could modulate inflorescence development. We have moved the images of the homozygous T3 plants to supplemental figure 2, and generated a new figure 3 with different T1 plants/lines representative of the different phenotypic categories after dexamethasone treatment. We have also included in the text the percentage of T1 lines belonging to each category. The phenotypes observed in homozygous T3 lines were stronger than the observed in heterozygous T1, where the senescence phenotype was rarely observed.

5) Manuscript length
At 700 lines of text, the manuscript seems rather long for the amount of primary data being presented. The section from lines 413-466 , describing the results of the transcriptomic analysis, could be dramatically condensed, since the justification of ABA as a target for further investigation does not need 50 lines of text, given the well-described role of HB21/40/53 in regulating ABA.

Paper extension is under the threshold length allowed by the journal. We decided to extend this section to show the relation between the AP2 regulation and HB21, in order to identify genes acting downstream the FUL-AP2-HB21 route instead of mis-expressed genes by *HB21* induction with no role in inflorescence arrest. For this reason, we would like to keep the RNAseq analysis section as it is.

Minor issues:
1) Line 61: "The flowering period is the time during which a plant produces flowers" this is rather a statement of the obvious.

We agree that the sentence is obvious, but we pretend just define the term to avoid misunderstanding. Unexpert readers could confuse flowering period with flowering time.

2) Line 66: "The inflorescence meristem, then, actively generates flowers" --> The inflorescence meristem then actively generates flowers

Changed

3) Line 125/126: IM arrest is reversible in Ler, but not in Col-0 (Walker et al, 2023), so this statement needs to be qualified - it is not universally true.

Our experience clearly indicated that Col. can be reactivated by AP2 induction (Balanzà et al., 2018) or fruit pruning, despite the response is weaker than in L.er. and we think that it can be affected by environmental factors or growing conditions. In fact, we have observed it many times in our growth conditions, and an example is also presented in this work in supplemental figure 1. It is possible that reactivation it is not universally true, we have not analyzed all ecotypes, but it seems likely, although variable phenotypically. Even if no visible new buds form (as in Walker et al., 2023) in some conditions, marker response (WUS expression, for example) is clearly reinstalled in the meristem upon fruit removal in all backgrounds we have analyzed, so we think this sentence should not be changed.

4) Line 138: " While the role of ABA in axillary bud dormancy is well established," -- I'm not sure that the role of ABA in axillary bud dormancy is really that well established (it is a very mild effect at best), but either way, this statement need supporting with references.

We have added the following references: Yao and Finlayson, 2015, González-Grandío et al., 2017 and van Es et al., 2024.

5) Line 144, Line 253: "associated to" --> associated with

Changed

6) Line 108, Line 176, Line 179, Line 181, Line 200, Line 211: In all of these places, the inflorescence meristem is instead referred to as a shoot apical meristem (SAM). This is technically correct, but confusing, because "inflorescence meristem" is used elsewhere for the same structure, and by convention, SAM usually refers to vegetative meristems. In would be better to use IM throughout.

The suggestion has been adopted.

7) Line 200: "where is no longer" --> "where it is no longer"

Changed.

8) Line 249: dexamethasone shouldn't have a capital letter, it isn't a proper noun.

Changed.

9) Line 299-301: ". This analysis indicated that the absence of HB21 could affect the number of flowers produced before arrest increasing the flower
production around 10%" -- the data don't really show this, because there is no statistical difference, and the range of the data is completely overlapping. I don't think this is a safe conclusion.

Text has been modified.

10) Line 305: "preliminary" --> previous

Changed.

11) Line 372: "doble" --> double
Changed.

12) Line 408, Line 559 : "Mann Whitney U's test" --> Mann Whitney U test

Changed.

13) Line 411: "arrest controlling" --> arrest by controlling

Changed.

14) Line 482-484: I think this is primary data and should be in a main figure, not in a supplemental.

As we show in figure 7A the accumulation of ABA in physiological conditions and its dependence on HB genes presence, we think it can be maintained in supplemental data.

15) Line 508: "significative" --> significant

Changed.

16) Line 524 and line 526: "respect to" --> with respect to

Changed.

17) "This outgrowth block mediated by BRC1 is highly dependent on ABA (van Es et al., 2023)". This is not what the data in van Es show -- indeed, they show that the effect of BRC1 is not highly dependent on ABA.

Text has been modified.

18) Line 612: "afecting mainly to flower development" --> mainly affecting flower development

Changed.

19) Line 614-615: there is evidence that CK specifically counteracts the repressive effect of ABA in bud outgrowth. In fact, CK represses BRC1, and therefore indirectly represses ABA and many other BRC1-regulated processes. This sentence needs changing.

We just wanted to state that ABA and CK are playing opposite roles, and we did not want to discuss about how CK counteracted ABA function. We have added a sentence to include the point of the referee.

20) Line 639-640: The data in Walker et al, 2023 show that floral arrest and inflorescence meristem arrest are indeed processes that occur close together in time (Figure 1A). It takes a long time for the effects of floral arrest to become visible, but the process is clearly initiated much earlier. However, the IM arrest and floral arrest processes do not seem to be completely synchronous.

We think we are proposing the same, that floral arrest and meristem arrest are not completely synchronous. In Walker et al, the determination of inflorescence arrest timing is calculated (not determined) based on macroscopic observations (basically cessation of bud initiation). However, we have used molecular markers in many of our studies, such WUS:GFP in this one or PIN:GFP in other studies, that show that it is not as early as calculated by Waleker et al, and probably the meristem is active but no longer bud initiation can be macroscopically observed. Here, we see how HB21 induction is almost synchronous with WUS:GFP repression, indicating that floral and inflorescence arrest could be coupled. However, these two things can be clearly uncoupled genetically (as in this study) or maybe by different growing conditions. Therefore, to clarify, we have modified slightly this sentence.

21) Line 667-675: This discussion of the possible relationship between arrest processes in cereals and Arabidopsis is certainly very interesting. However, it also represents a massive over-simplification. The arrest processes are very different between different cereals, and in wheat for instance, there is no pre-anthesis tip degeneration (instead, the spike meristem is converted to a spikelet meristem). This discussion needs far more space that it can be given here, since it also needs actually descriptions of the shoot architecture for different cereals in order for the reader to understand it). So I would suggest dramatically shortening this paragraph instead, and just noting that Grassy Tillers has been implicated in the end of flowering in barley, without going into any of the morphological detail.

Referee is right and we over-simplificate the comparison considering all cereals. We have now focused the discussion in barley specifically.

Reviewer #2 (Comments for the Author):

In this manuscript, Sánchez-Gerschon et al. aimed to understand the molecular mechanism of inflorescence arrest at the end of flowering in Arabidopsis thaliana. The authors focused on HOMEOBOX PROTEIN 21/40/53 (HBs) as key regulators in the control of inflorescence arrest and showed that the three HBs play an important role in the control of inflorescence development through ABA biosynthesis and response. This manuscript will provide important information on how the inflorescence meristem arrests at the end of flowering.

Major comments:

1. The expression data shown in Figures 1 and 2 strongly suggest that HB21 is involved in inflorescence arrest. However, genetic analyses are not sufficient to support the authors' claim. To further clarify the function of HBs in inflorescence arrest, the authors should analyse the expression of WUS-GFP in HB21 ind plants (#21 and #7). Furthermore, the inflorescence phenotype of HB21 ind plants (#21 and #7) is not well characterized (Fig. 3 B/C). Alterations in inflorescence architecture should be more clearly described and discussed using quantitative data.

The main objective of figures 1 and 2 was to relate the function of HB21 to the control of the end of flowering, not to show a regulatory effect on WUS. In the inducible *HB21* lines we could observe, or not, an effect on *WUS,* but the study of this regulation is probably out of the scope of this paper and would conform a new and different paper. The genetic analysis, understood as the phenotypic characterization of the different mutants presented in the work, clearly supports the role in the control of the inflorescence arrest by the *HB* genes trough the ABA regulation, as mutants develop more flowers that the control plants.

Respect to figure 3, our intention was to provide qualitative data, more than quantitative, about the putative role of HB21 in the control of inflorescence arrest. We have moved the images of the homozygous T3 plants to supplemental figure 2, and generated a new figure 3 with different T1 plants/lines representative of the different phenotypic categories after dexamethasone treatment. We have also included in the text the percentage of T1 lines belonging to each category. The phenotypes observed in homozygous T3 lines were stronger than the observed in heterozygous T1, but all indicating an effect in floral bud or inflorescence development.

2. With regard to the HB 40 and HB53 genes, the spatial expression patterns of these genes in the inflorescence should be analysed using a GUS reporter as well as HB21 (González-Grandío et al, 2017). In the discussion part, the authors propose the possibility that the transcripts of the HB genes directly affect the activity of the

meristem in a non-cell autonomous manner. The expression data for HB 40/53 in the inflorescence may be helpful in supporting the authors' claims.

We originally tried to check also the *HB40* and *HB53* reporter lines suggested, but the signal of those reporters were weak and appear to be silenced at the end of the flowering cycle (as it could be deduced by the inconsistency of the lack of GUS activity in arrested inflorescences and the upregulation of HB40 and HB53 that we found by RT-Q-PCR), so we favor to show the analyses of the expression by RT-Q-PCR.

3. The three HB genes mentioned in this paper are up-regulated in axillary meristems by the TCP transcription factor BRC1. Are BRC1 or other TCPs involved in the transcriptional activation of HBs in the inflorescence shoot and in inflorescence arrest at the end of flowering? Since we don't know how environmental signals induce the HB genes, if known, it would be good to state.

HB genes can be activated at the end of flowering in an independent way of BRC1. Our data indicates that AP2 is a clear and direct activator of these genes. We don't have evidence that *BRC1* or *BRC2* are expressed in inflorescence meristems at the end of flowering (Wüest et al., 2016; Martínez-Fernández et al 2020). In this work, we have not explored a putative role of BRC1 or other TCPs in proliferative arrest, but certainly it would be interesting to check in the future.

Minor comments:
There are many typographical errors (L170, 186, 189, 229, 315, 366, 368, 487, 488, 489, 490, 538, 633, 696 etc.) in this manuscript. Please correct them in the revised version.

Text has been updated and checked for typographical errors.

01-Apr-2024
Dr. Vicente Balanzà
Instituto de Biologia Molecular y Celular de Plantas
Development
Valencia 46022
Spain

MSID: PP2023-RA-01998R1
MS TITLE: HB21/40/53 promote inflorescence arrest through ABA accumulation at the end of flowering

Dear Dr. Vicente Balanzà:

I am pleased to inform you that your revised manuscript "HB21/40/53 promote inflorescence arrest through ABA accumulation at the end of flowering" has been accepted for publication in Plant Physiology.

This paper is accepted, but as reviewer 2 has suggested, I hope that you will improve the phenotypic data and apply what you will find to your future research.

Your manuscript will be checked for consistency with journal formatting and image standards by one of our Science Editors, who will contact you shortly. They may have a number of queries and suggestions for you. Please keep in mind that they work to increase the online visibility of your article and its attraction for readers from the widest of backgrounds. You will have the opportunity to review any revisions, and I hope you will work constructively with them to get the most out of your research. You will also have the opportunity to review all changes when you receive the author proofs for your article.

Once validated for publication, your article will be sent to a Science Editor for a final review of your manuscript and source files. After your final files are uploaded, your article will appear in the next available issue and the online Preview version of your article will be posted after your signed Copyright and Checklist forms and final source files are received.

Please be sure to include the following in your manuscript file:
Harvard style citations
List of all author contributions and funding information at the beginning of manuscript after the titles and author list.
List all supplemental materials associated with the article, with titles, at the end of the ms, before the references and acknowledgments section.
Complete Figure Legends for all figures appearing in the article listed before the Literature Cited.

Thank you and congratulations on your Plant Physiology paper!

Sincerely,
Plant Phys Board of Editors

=====
FIRST AUTHOR PROFILES
=====

We recognize the hard work that goes into being First Author of a Plant Physiology paper by publishing a "First Author Feature" for each First Author alongside your paper.

If the first author(s) would like to submit information for a feature on Facebook and Twitter, fill out the form by clicking on the link below:

Plant Physiology First Author Feature Form: https://forms.office.com/r/ZBCGZfhm8F
If you cannot access the form through the link above, please contact Rachel at rbelsky@aspb.org.

To upload an image of yourself to accompany your profile, please put your photo in the correct journal folder at the link below. You must label your image: Author First Name Last Name Article Title.

First Author Profile Image Dropbox: https://www.dropbox.com/scl/fo/xz029p7qjr2jml2rp2kmq/h?dl=0&rlkey=6fkxcaqmxx35ap152b2osy1fz

=====
IMPORTANT REMINDER: PEER REVIEW REPORTS
=====

If you opted to publish a peer review report along with your article during the original submission process, it will be prepared by the editorial staff and publicly posted with your manuscript, inside the zip file that contains any other supplemental material. As a reminder, the peer review report is a public record of all comments from editors and reviewers, as well as your prior responses, as you received them in the decision letters for each draft of your manuscript. If you agreed to publish this report and have changed your mind, or are not sure if you selected this option, please contact the editorial office as soon as possible before signing the license agreement from our publisher.

-- Reviewer comments:
Reviewer #1 (Comments for the Author):

In this revised version of the manuscript, I believe the authors have addressed all my major points of concern, or have justifiably rebutted my comments. Overall, I think this is an excellent piece of work, which is now presented more accurately and clearly. I don't have any outstanding concerns.

Reviewer #2 (Comments for the Author):

The authors do not seem to have responded sincerely to the reviewer's comments, but I have decided to give a positive evaluation of the inflorescence phenotype of the HB21 ind T1 plants, which has been added as a new Figure 3. In the new Figure 3, a common phenotype was observed in several HB ind T1 lines, although quantitative analysis is not yet sufficient. A more detailed analysis of the expression of HB 21 and a phenotypic analysis of the HB21 ind lines will be required for a convincing paper.
